# Hydroclimatic contrasts over Asian monsoon areas and linkages to tropical Pacific SSTs

**DOI:** 10.1038/srep33177

**Published:** 2016-09-09

**Authors:** Hai Xu, Jianghu Lan, Enguo Sheng, Bin Liu, Keke Yu, Yuanda Ye, Zhengguo Shi, Peng Cheng, Xulong Wang, Xinying Zhou, Kevin M. Yeager

**Affiliations:** 1 State key Laboratory of Loess and Quaternary Geology, institute of Earth Environment, Chinese Academy of Sciences, Xi’an, China; 2 Department of Environment Science and Technology, School of Human Settlements and Civil Engineering, Xi’an Jiaotong University, Xi’an, China; 3 Laboratory of Human Evolution and Archeological Science, Institute of Vertebrate Paleontology and Paleoanthropology, Chinese Academy of Sciences, Beijing, China; 4 Department of Earth and Environmental Sciences, University of Kentucky, Lexington, KY 40506, USA

## Abstract

Knowledge of spatial and temporal hydroclimatic differences is critical in understanding climatic mechanisms. Here we show striking hydroclimatic contrasts between northern and southern parts of the eastern margin of the Tibetan Plateau (ETP), and those between East Asian summer monsoon (EASM) and Indian summer monsoon (ISM) areas during the past ~2,000 years. During the Medieval Period, and the last 100 to 200 years, the southern ETP (S-ETP) area was generally dry (on average), while the northern ETP (N-ETP) area was wet. During the Little Ice Age (LIA), hydroclimate over S-ETP areas was wet, while that over N-ETP area was dry (on average). Such hydroclimatic contrasts can be broadly extended to ISM and EASM areas. We contend that changes in sea surface temperatures (SSTs) of the tropical Pacific Ocean could have played important roles in producing these hydroclimatic contrasts, by forcing the north-south movement of the Intertropical Convergence Zone (ITCZ) and intensification/slowdown of Walker circulation. The results of sensitivity experiments also support such a proposition.

Knowledge of climatic changes that occurred over the past 1,000 to 2,000 years is crucial to understand contemporary climate dynamics, and to effectively predict possible climatic changes in the future. Numerous achievements have been made in reconstructing the general trends of global climatic change during this time period (e.g., ref. 1 *and references therein*), which have greatly improved our understanding of climate changes and the roles of various natural and anthropogenic climatic forcing mechanisms. In recent years, an increasingly large body of evidence has shown clear climatic discrepancies on different time scales between different regions^2^,^3^,^4^,^5^. For example, hydroclimatic patterns during the Medieval Period (~800–1300 AD) vary between different regions of the world^2^. Climatic changes during the Little Ice Age (LIA; ~1400–1800 AD) and those of the past 200 years have also been reported to be variable between different regions^3^,^4^. However, the causes of such regional hydroclimatic differences are poorly known.

The Asian summer monsoon system is generally divided into two subsystems, namely the Indian Summer Monsoon (ISM), and the East Asian Summer Monsoon (EASM) (e.g., ref. 6 and Fig. 1), both of which play key roles in transporting heat and moisture from tropical oceans to higher latitudes. The ETP area is one of the most sensitive regions for studying the behavior of these two monsoons and their linkages to global climatic changes. Along the ETP, there prevails the EASM, the ISM, the East Asian winter monsoon, and the westerly jet stream^3^,^6^. Our previous work has shown clear climatic discrepancies on a decadal time scale during the past ~400 years between northern ETP (N-ETP) and southern ETP (S-ETP) areas^3^. A recent study compared palmer drought severity indices (PDSI) over the north and south Tibetan Plateau developed from tree ring widths during the past ~550 years, and showed two obvious contrasts in moisture stress between these two regions^5^, suggesting that the responses of climatic changes are sensitive and variable to different climatic forcing over the eastern Tibetan Plateau. Here, we focus on centennial/multi-decadal hydroclimatic differences along ETP areas during the past 2,000 years, then extend such hydroclimatic contrasts to the broader EASM and ISM areas, and finally we discuss possible forcing mechanisms.

## Climatic Changes Over N-ETP Areas

Climatic changes over N-ETP areas are well-synchronized on centennial/multi-decadal time scales (Fig. 2). For example, the long term trends in precipitation at Lake Qinghai inferred from sedimentary C/N ratios and grain size^7^ (see details in Supplementary 1) are similar to those reconstructed from tree ring widths at Qilian Mt.^8^, Dulan^9^ (Fig. 2), and Delingha^10^ (Fig. S3), and similar to the trend of drought/flood index derived from historical literature^11^.

Precipitation over N-ETP areas was clearly higher during the interval of ~750–1200 AD, corresponding to a wet Medieval Period. This hydroclimatic feature can be discerned from both Lake Qinghai sediments, and from nearby tree ring records (e.g., at Qilian Mt.^8^ and Dulan^9^). During the Medieval Period, higher precipitation led to enhanced surface runoff, which resulted in an increased influx of coarser sediments, as reflected in sediment grain size data from Lake Qinghai (Fig. 2). Increased sediment C/N ratio values, and decreased δ^13^C values of the bulk organic matter (δ^13^C_org_) also indicate that higher amounts of terrestrial organic matter were transported into the lake during the Medieval Period (See Supplementary 1 and Fig. S2 for details).

The average hydroclimatic conditions during LIA were drier over the N-ETP, as inferred from tree ring records at Qilian Mt.^8^ and Dulan^9^. The sediment grain sizes at Lake Qinghai were considerably smaller during the LIA, indicating weaker surface runoff during this period. C/N ratio values, total organic carbon content (TOC), and δ^13^C_org_ values from Lake Qinghai sediment (Fig. S2) also indicated dry conditions.

Precipitation during the past 100 to 200 years showed an increasing trend, as inferred from sediment grain size data from Lake Qinghai (Fig. 2). This trend is also similar to those developed from tree rings at Qilian Mt.^8^ and Dulan^9^, and similar to the trend of drought/flood indices from the Longxi area derived from historical literature^11^.

## Climatic Changes Over S-ETP Areas

Climatic changes over S-ETP areas are broadly anti-phased with those over N-ETP areas (Figs 2 and 3). One striking feature is the drought during the Medieval Period. For example, conifer pollen concentrations in Lake Erhai sediments decreased appreciably during the Medieval Period, suggesting dry climatic conditions^12^. The sediment grain size at Lake Lugu also decreased during this time (Fig. 3), suggesting weakened surface runoff intensity^13^. Both TOC content and C/N ratio values of sedimentary organic matter from Lake Lugu decreased appreciably as well, suggesting decreased influx of terrestrial organic matter due to decreased monsoon precipitation during this period^13^.

The LIA in S-ETP areas was relatively wet as inferred from limnological records (Fig. 3), which contrasts with the arid conditions in N-ETP areas during this time. For example, herb and conifer pollen concentrations in Lake Erhai sediments increased during this interval (Fig. 3)^7^,^12^. Sediment grain size at Lake Lugu increased as compared with those both before and after the LIA^13^ (Fig. 3). C/N ratio values of sedimentary organic matter at Lake Lugu also increased (Fig. 3), suggesting increased contributions of terrestrial organic matter during the LIA^13^.

Wet climatic conditions during the LIA in S-ETP areas can also be inferred from historical literature, and from variations in lake levels at Lake Chenghai. For example, historical literature recorded that the lake level began to decrease in the middle of the Ming Dynasty (1368 to 1644 AD), and a dam was constructed across the Chenghe River to store water during the Wanli Empire (1573–1620AD)^14^ (Supplementary 1). The dam was repeatedly rebuilt, and the river channel was widened several times during the Qing Dynasty (1636 to 1912 AD) due to lake level fluctuations as recorded in the *New Yunnan chorography*^14^ (Supplementary 1). These historical records indicate that there were some intervals (within the LIA) during which lake levels were considerably higher than today (see details in Supplementary 1). Radiocarbon ages of aquatic snail shells buried in the outcrops/shorelines around Lake Chenghai indicate several low stands in lake levels during the Medieval Period (Fig. S4 and Table S2). Taken together, the historical literature and the outcrop/shoreline evidence suggest that lake levels were relatively low during the Medieval Period, but higher during the LIA at Lake Chenghai.

Precipitation in S-ETP areas clearly decreased during the past 100 to 200 years (Fig. 3), which is the reverse of that in N-ETP areas (Fig. 2). For example, the sediment grain size and C/N ratio values at Lake Lugu suggest a clear decrease in monsoon precipitation during the past 100 to 200 years (Fig. 3). A sharp decrease (by ~33 meters) in lake level at Lake Chenghai occurred during the 44^th^ year (corresponding to 1779 AD) of the Qianlong Empire (1711–1799 AD), Qing Dynasty, as recorded in the chorography of *Yong Bei Zhi Li Ting Zhi*^15^, and Lake Chenghai switched from a hydrologically open lake to a closed one thereafter^7^, which also supports decreasing ISM precipitation during the past ~200 years. Such decreasing ISM precipitation trends have also been captured by a large number of proxy indices, including tree ring and ice core records (see ref. 16 and references therein).

## Medieval Hydroclimatic Contrasts Between EASM and ISM Areas

**Figure 1:**
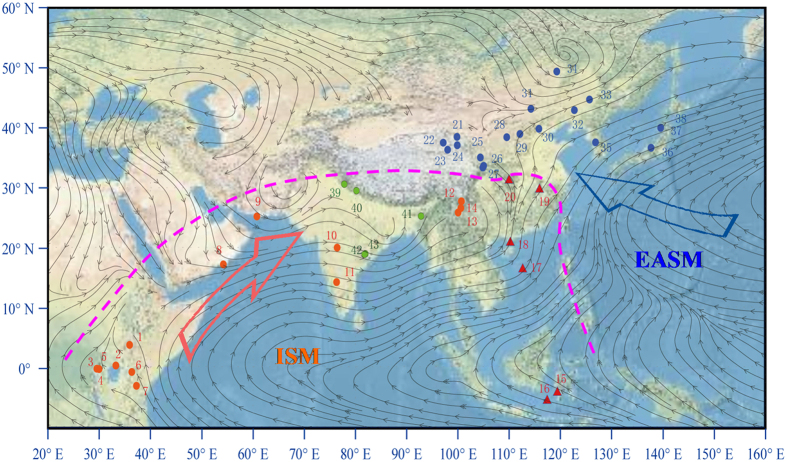
Hydroclimatic contrasts between EASM and ISM areas. Arrows denote monsoon streamlines (for areas lower than 1500 m) averaged from June to August at 850 hPa during 1968–1996 based on NCEP/NCAR reanalysis data^61^. The blue and orange circles show wet and dry sites during the Medieval Period, respectively. The red triangles denote the sites (15 to 20) located in an ISM-EASM transitional zone. Green circles (39 to 43) show the locations of some stalagmite records in the northern India. The dashed pink line outlines the general position of the ISM boundary. Numbers denote the sites mentioned in the text (note parts of the sites are overlapped; see details in Table S3). The satellite image was drawn from the basemaps in ArcGIS 10.2 (ESRI data & maps).

**Figure 2:**
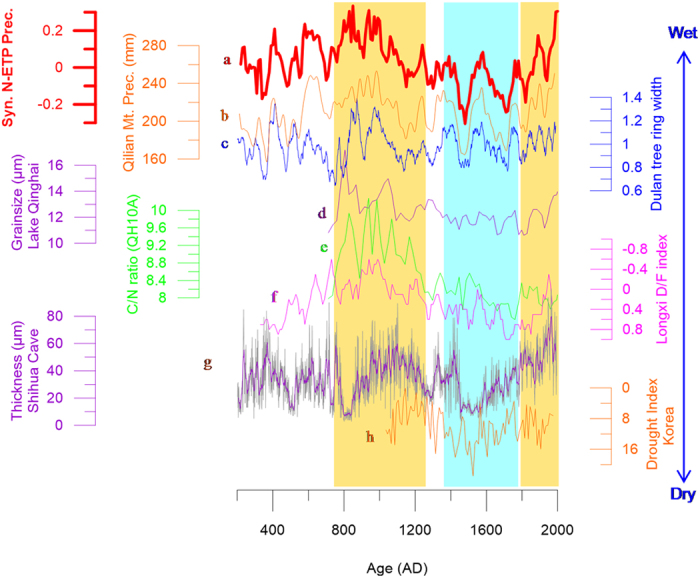
Comparison of hydroclimatic changes in N-ETP and EASM areas. (a) (red) is composited precipitation in N-ETP areas (see Supplementary 1 for details). (b) (orange) is precipitation reconstructed from tree ring width at Qilian Mt.^8^ (c) (blue) is the Dulan tree ring width data^9^. (d) (purple) and (e) (green) denote the grain size and organic matter C/N ratio values in Lake Qinghai sediments^7^. (f) (magenta) is the drought/flood index at Longxi inferred from historical literature^11^. (g) (purple) is the lamina thickness of a stalagmite in Shihua Cave^22^. (i) (orange) is the drought index at Seoul, South Korea^27^. See the comparison sites in Fig. 1 and Table S3. The yellow shaded columns indicate the Medieval Period and the last ~200 years, and the blue shaded column indicates the LIA.

Because precipitation in S-ETP areas is controlled by the ISM^7^,^12^,^16^,^17^, and that in N-ETP areas is likely dominated by the EASM on centennial/decadal time scales during the past 2,000 years (see Supplementary 2), it is necessary to examine whether the hydroclimatic contrasts between S-ETP and N-ETP areas can be extended to EASM and ISM areas. Previous studies indicate that there prevailed “warm-wet” Medieval climates at a large number of sites (see blue sites in Fig. 1) over the broad area from northern China to central and northern Japan, generally referred to as the EASM region (e.g., ref. 6). For example, pollen data from the Maili peat indicates that the Horqin sand lands in northeastern China were much wetter during this period^18^. Paleosols, fluvial and lacustrine sediments indicate that from about 1000–1400 AD, precipitation in the Otindag Desert of inner Mongolia was much higher than at present^19^. Several lines of evidence suggest that much warmer and wetter climatic conditions prevailed over extensive areas of sand lands in eastern China during the Medieval Period, including the Maowusu, Songnen, Hulunbeier and Keerqin sand lands^19^,^20^ (see locations in Fig. 1). The climate in northern Shanxi Province, China, as recorded in the sediments of Lake Gonghai (Fig. 1), was also warm and wet during Medieval times^21^. The thicknesses of stalagmite lamina at Shihua Cave also indicate higher precipitation during the Medieval Period than during the LIA^22^ (Figs 1 and 2). Evidence from Chinese historical literature also documents more favorable climatic conditions during the Medieval Period. For example, the northern boundaries of some plants, including citrus, Chinese grass (*Boehmeria nivea*), wheat, sugarcane, and tea, were farther to the north during the Medieval Period than they are today^23^. The northern boundaries of the subtropical and temperate zones were approximately 1° farther north than their present locations^24^. Warm and/or wet climatic intervals during the Medieval Period have also been reported in Japan and Korea. For example, it was warm and wet during the Medieval Period as inferred from multiple sedimentary records at Lake Nakatsuna (central Japan; Fig. 1)^25^, and at Lakes Ni-no-Megata and San-no-Megata (northeastern Japan; Fig. 1)^26^. The drought index inferred from historical literature at Seoul, South Korea also clearly indicates wetter conditions during the Medieval Period than those during the LIA^27^.

**Figure 3:**
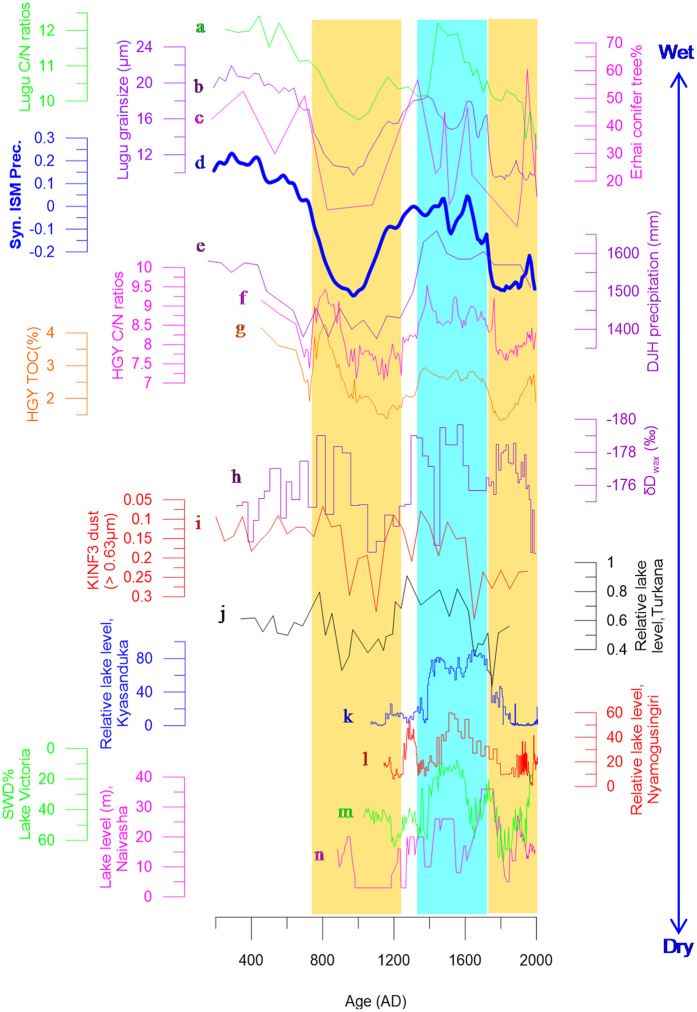
Comparison of hydroclimatic changes in S-ETP and ISM areas. (a) (green) and (b) (blue purple) denote C/N ratio values and grain size of lake sediments from Lake Lugu^13^. (c) (magenta) is the conifer tree pollen concentration (%) at Lake Erhai^12^;)(d) (thick blue line) is the composited precipitation over S-ETP areas (see Supplementary 1 for details). (e) (purple) is precipitation at Lake Dajiuhu^31^. (f) (magenta) and (g) (orange) denote C/N and TOC% ratio values from sediments of Lake Huguangyan^29^. (h) (purple) denotes δD_wax_% in marine sediments at Makassar Strait^34^. (i) (red) is Kilimanjaro ice core dust content^42^. (j) to n denote changes in lake levels (or lake level indicators) at Lakes Turkana (black)^39^, Kyasanduka (blue)^41^, Nyamogusingiri (red)^41^, Victoria (green)^40^, and Naivasha (magenta)^38^, respectively. See the comparison sites in Fig. 1 and Table S3. The yellow shaded columns indicate the Medieval Period and the last ~200 years, and the blue shaded column indicates the LIA.

Conversely, a considerable body of evidence indicates dry conditions in most ISM areas and/or in the ISM-EASM transitional zone (see red sites in Fig. 1). For example, the sharp decreases in organic matter contents in a peat core from the Zoige Plateau, mid-ETP^28^, suggest a dry hydroclimatic condition during the Medieval Period. Such Medieval droughts have also been reported at Lake Huguangyan, in southern China^29^ (Figs 1 and 3), and at Lake Longgan in the lower reaches of the Yangtze River (Fig. 1)^30^. Dry climatic conditions during the Medieval Period can also be seen from pollen records in peat from the Dajiuhu area, Hubei province, mid-southern China (Figs 1 and 3)^31^. Climate over the South China Sea^32^ and the Western Pacific warm pool (WPWP)^33^,^34^ were also much drier during the Medieval Period (see locations in Fig. 1). For example, the hydrogen isotopic ratios of terrestrial higher plant leaf waxes (δD_sw_) in sediments from cores 31MC and 34GGC collected from the Indo-Pacific warm pool^34^ indicated a much drier Medieval Period (Fig. 3). The sites from 15 to 20 (Fig. 1) are generally recognized to be located within an ISM-EASM transitional zone, as inferred from modern meteorological records^35^,^36^,^37^. However, more precipitation over the transitional zone is likely to originate from the Indian Ocean^37^. The hydroclimatic patterns of the sites (15 to 20) are also more similar to those over ISM areas during the Medieval Period (as mentioned above; Fig. 3), but are broadly in contrast to those over EASM areas (Fig. 2), suggesting that the medieval drought over the ISM areas could have extended to a much wider area during this period.

In eastern Africa, the water level of Lake Naivasha dropped markedly during the Medieval Period, and the lake water salinity increased concurrently^38^ (Figs 1 and 3). The water level at Lake Edward, inferred from shallow water diatom content (SWD%; Figs 1 and 3) was also much lower during the Medieval Period than during the LIA^39^. Water levels at Lakes Turkana^39^, Victoria^40^, Kyasanduka and Nyamogusingiri^41^ were all lower during the Medieval Period than during the LIA (Figs 1 and 3). Ice core records from Mt. Kilimanjaro also show a clear decrease in monsoon intensity during this period^42^ (Figs 1 and 3). A recent study^43^ found clear evidence for a medieval drought at Oman Gulf and neighboring areas, as inferred from changes in vegetation types. During the Medieval Period, the vegetation in southern Iran was dominated by desert taxa, including *Amaranthaceae, Caryophyllaceae, Asteraceae, Centaurea and Calligonum*. In parallel, in the Gulf of Oman, the presence of *Impagidinium paradoxum* indicates a lack of freshwater discharge into the ocean around this time. All these lines of evidence suggest a clear hydroclimatic difference between EASM and ISM areas during the Medieval Period.

## LIA Hydroclimatic Contrasts Between EASM and ISM Areas

The LIA’s dry climatic conditions extended over a large area from northern China to central-northern Japan (e.g., refs 11,21,25,26 and 44). For example, δ^18^O data from stalagmites in Wanxiang Cave^44^ and Huangye Cave^11^ indicate dry LIA conditions. Precipitation inferred from sedimentary pollen records and magnetic characteristics at Lake Gonghai, northern China was much lower during the LIA^21^. Sandlands were widespread in northern China during this period^20^.

Conversely, wetter climatic conditions existed in S-ETP areas as inferred from limnological evidence at Lakes Erhai^12^, Lugu^13^, and Chenghai, China (Supplementary 1). The transitional sites 15 to 20 again show similar hydroclimatic patterns with those of the ISM areas (but in contrast with those over EASM areas). For example, C/N ratio values in sediments from Lake Huguangyan increased significantly during the LIA (Fig. 3 and ref. 29), suggesting increasing precipitation. Wet LIA climatic conditions can also be inferred from pollen records preserved in peats from the Dajiuhu area^31^. Precipitation over the tropical western Pacific Ocean increased during this period, as inferred from oceanic/lacustrine records (e.g., refs 32, 33, 34). The wetter LIA climatic conditions can also be extended to eastern Africa. For example, lake water levels at several lakes clearly increased (Figs 1 and 3), e.g., Lakes Naivasha^38^, Turkana^39^, Edward^39^, Victoria^40^, Nyamogusingiri and Kyasanduka^41^, suggesting increased precipitation during the LIA. At the Oman Gulf, a sudden increase in *Spiniferites ramosus* occurred at ~1440 AD; increased freshwater input into the ocean can also be inferred at this time, suggesting a relatively wetter climate during the LIA, with an increase in ISM intensity^43^. All these lines of evidence suggest clear hydroclimatic contrasts between the ISM and the EASM during the LIA.

## Recent Hydroclimatic Contrasts Between EASM and ISM Areas

Hydroclimatic contrasts between S-ETP and N-ETP areas during the past 100 to 200 years are very similar in pattern with those during the Medieval Period; and such climatic differences also existed between ISM and EASM areas. For example, several lines of evidence show a long-term decreasing trend in precipitation in both S-ETP^3^,^16^ and ISM areas^32^,^34^. Meanwhile, precipitation during the past 100 to 200 years has been increasing in N-ETP areas (e.g., refs 8, 9, 10), and over a wide geographic extent in EASM areas (e.g., refs 7,11,21 and 44). Recently, Hong *et al*.^45^ showed clear hydroclimatic contrasts over the Chinese mainland and adjacent areas during the Younger Dryas. Although the spatial and temporal scales of the hydroclimatic contrasts of Hong *et al*.^45^ are more or less different than those concerned in this study, it shows again that the hydroclimatic changes between the broad ISM and EASM areas are quite different.

## Hydroclimatic Contrasts Between ISM and EASM areas: Possible Mechanisms

The ITCZ and El Niño Southern Oscillation (ENSO) are widely considered the two most important phenomena influencing Asian summer monsoon intensities. The northern movement of the mean position of the ITCZ is generally linked to an intensified EASM since ‘*the summer monsoon is the north frontier of ITCZ*’^46^. As the precipitation zone moves northward, less precipitation would be delivered to the southern tropical Pacific Ocean^47^, and to most ISM areas, including eastern Africa, southern China, the South China Sea, and the extended WPWP areas^33^,^34^. Conversely, the southern movement of the ITCZ may lead to less precipitation in most EASM areas, but increased rainfall over the southern tropical Pacific Ocean (e.g., refs 33 and 47), and most ISM areas. This implies that the north-south movement of the ITCZ may produce a broadly inverse relationship between precipitation over EASM and ISM areas. Tierney *et al*.^34^ showed an inverse relationship between the EASM and the Indonesian monsoon, and they suggested that the movement of the ITCZ and the associated changes in monsoon intensity were responsible for such an inverse relationship.

The relationship between ENSO and ISM precipitation has also been widely studied^48^. Higher SSTs in the eastern tropical Pacific Ocean (e.g., SST_Niño3–4_) correspond to decreases in ISM intensity, and vice versa^48^. One possible interpretation is that when SSTs in the eastern tropical Pacific Ocean increase (El Niño status), the zonal SST gradient in the tropical Pacific Ocean may decrease, which would lead to a decrease in Walker circulation, followed by a decrease in ISM intensity^49^. On the other hand, when SSTs in the eastern tropical Pacific Ocean decrease (La Niña status), the zonal SST gradient there increases, and Walker circulation may be intensified, which would result in an intensified ISM.

**Figure 4:**
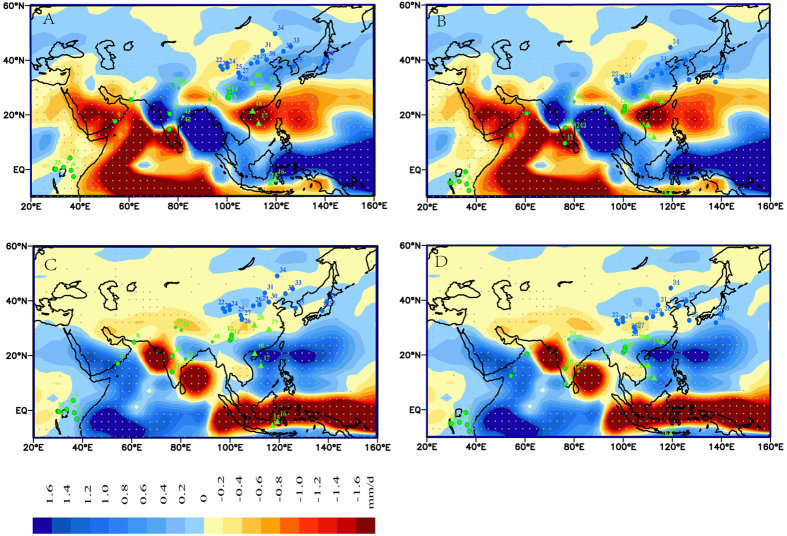
Responses of monsoon precipitation to changes in tropical Pacific Ocean Sea surface temperatures (SSTs). (**A**,**C**) show the results of 1 °C warming and cooling experiment, respectively. The sites in A and C are similar to those in Fig. 1 except that all of the sites in ISM areas are changed to green (for a better color contrast). (**B**,**D**) are similar to A and C except that all of the sites are moved 5° southwards. The legend shows changes in precipitation (mm/d). Dotted areas represent significant levels higher than 95%. The simulations and the basemaps were drawn in Grid Analysis and Display System (GrADS) 1.9. See details of the sensitivity experiments in Supplementary 3.

We suspect that changes in SSTs in the tropical Pacific Ocean may have played an important role in generating the large-scale hydroclimatic contrasts between EASM and ISM areas (and thereafter between the N-ETP and S-ETP areas), through the migration of the ITCZ and intensification/weakening of Walker circulation. We simulated precipitation changes over Asian monsoon areas under warming and cooling scenarios over different regions of the tropical Pacific Ocean (see details in Supplementary 3), and the results show that under a scenario of 1 °C warming (Fig. 4A) and cooling (Fig. 4C) over the entire tropical Pacific Ocean, hydroclimatic changes between ISM and EASM areas do show clear contrasts. For example, under the warming scenario, precipitation in northern and northeastern China, Korea, and Japan increases, while precipitation in most ISM areas decreases, including eastern Africa, the western and northern Indian Ocean, and most WPWP areas (Fig. 4A). While under a scenario of 1 °C decrease in SST, precipitation in the western Indian Ocean and WPWP areas increases markedly, but that in EASM areas is less clear (Fig. 4C).

We acknowledge there would be certain uncertainties in the model output (especially from a simple simulation as described in Supplementary 3), and we also note that the modern hydroclimatic contrasts (model simulation in this study) are somewhat different with those inferred from geological evidence during the Medieval Period and LIA. However, if we move the locations of the paleoclimatic records 5° southwards (or move the model output 5° northwards), the simulated hydroclimatic contrasts of the warming and cooling experiments correlate considerably well with those observed in paleoclimatic records during the Medieval period (Fig. 4B) and LIA (Fig. 4D), respectively. This further suggests that systematic hydroclimatic contrasts do exist between ISM and EASM areas, and such hydroclimatic contrast patterns are also meridionally variable as a function of time.

It is interesting to note that precipitation in northern India and the Bay of Bengal show very different signals as compared with those of most other ISM areas, both in the warming (Fig. 4A) and cooling simulations (Fig. 4C). Such spatial differences in precipitation have also been observed both in meteorological and paleoclimatic records (e.g., refs 50 and 51), and are possibly related to the movements of monsoon troughs in southern Himalayan and southern Asian areas^52^. In addition, it is likely that water vapor transported from the northern Bay of Bengal to northern India^53^,^54^,^55^ may also compete with that from the northern Arabian Sea and further complicate the precipitation signal over those areas. Meanwhile paleo-hydroclimatic patterns over central to southern India, where water vapor is mostly from the Arabian Sea, are broadly similar to those over the ISM areas as mentioned above. For example, at Lake Lonar (Fig. 1), central India, the Medieval Period was much drier than the LIA as inferred from multi-proxy indices^56^. Lake level variations in a southern India lake (Thimmannanayakanakere; Fig. 1) also showed much higher lake levels during the LIA as compared with those during the Medieval Period^57^.

### Oceanic warming

Cobb *et al*.^58^ suggested a possible colder status (analogous to a prolonged La Niña/La Niña-like status) in the eastern tropical Pacific Ocean during the Medieval Period based on coral δ^18^O data. However, in recent years, increasing biogeological evidence supports a warmer status of the tropical eastern Pacific Ocean during the Medieval Period (see Supplementary 4 for details). On the western side of the tropical Pacific Ocean, reconstructed SSTs over WPWP areas show that it was warmer during the Medieval Period than those before and after (e.g., ref. 59 and Fig. S6). Since modern observations show that SSTs in WPWP areas are broadly synchronous with those in the eastern tropical Pacific Ocean (e.g., ref. 60), it is likely they were also synchronous during the Medieval Period. This again suggests the possibility of a warmer Medieval Period in tropical Pacific Ocean areas. If the whole tropical Pacific Ocean was warmer during the Medieval Period, this would have led to northern movement of the ITCZ, and an intensified EASM, but declined ISM (similar to the results of the warming experiment shown in Fig. 4A).

Although it remains unclear what drove the medieval warming in the tropical Pacific Ocean, it is reasonable that SSTs in different regions of the tropical Pacific Ocean responded differently to similar external/internal forcings because of different background SSTs. Increases in SSTs in the eastern tropical Pacific Ocean are expected to be relatively higher (in percentage) under an external/internal forcing because the background SSTs over eastern tropical Pacific are much lower as compared to those over western tropical Pacific. Providing this scenario, zonal SST gradients in the entire tropical Pacific Ocean would be decreased (analogous to a prolonged El Niño or El Niño-like status), which would weaken Walker circulation, and eventually lead to decreased ISM intensity during the Medieval Period. For example, the medieval droughts in southern China are broadly synchronous with the El Niño frequencies in tropical eastern Pacific Ocean areas (Fig. S6). Sun *et al*.^32^ also found decreased ISM precipitation in the southern China Sea during the Medieval Period, and they suggested that Walker circulation may have slowed during this period.

As mentioned, hydroclimatic contrasts during the modern epoch are similar to those during the Medieval Period as inferred from paleaoclimatic records. Both meteorological records and proxy indices show increases in SSTs in the tropical Pacific Ocean^59^,^60^, which would lead to northward movement of the ITCZ and reduced Walker circulation. Weakening of Walker circulation during recent times has also been reproduced in the simulation of Tokinaga *et al*.^49^. This means that both the northern movement of the ITCZ and the slowdown of Walker circulation during the modern epoch are similar to those during the Medieval Period, resulting in similar hydroclimatic patterns in Asian summer monsoon areas during these two periods.

### Oceanic cooling

Decreases in SSTs in the tropical Pacific Ocean during the LIA may have led to inverse scenarios to those during the Medieval Period, i.e., southern movement of the ITCZ and intensified Walker circulation (analogous to a prolonged La Niña or La Niña-like status), which would have led to decreased EASM intensity, but increased ISM intensity (Fig. 4B). Such a scenario is also supported by geological evidence. For example, Sachs *et al*.^47^ showed increased precipitation during the LIA at the Northern Line Islands, Galápagos and Palau, in the central tropical Pacific Ocean, and they suggested that the ITCZ may have moved 500 km southwards to its modern position. The wetter conditions in the Indo-Pacific warm pool areas during the LIA were also attributed to southern displacement of the ITCZ, as compared with its modern position^33^.

## Conclusions

We show striking hydroclimatic contrasts between N-ETP and S-ETP areas during the past ~2,000 years, and extended such hydroclimatic contrasts to ISM and EASM areas. During the Medieval Period, and the last 100 to 200 years, the S-ETP area and most ISM areas were dry, while the N-ETP area and EASM areas were wet. During the LIA, hydroclimate in the S-ETP area and most ISM areas were wet, while those over the N-ETP and EASM areas were dry. We propose that changes in SSTs in the tropical Pacific Ocean may have played important roles in producing such hydroclimatic contrasts through the north-south movement of the ITCZ and intensification/slowdown of Walker circulation. We conclude that (1) During the Medieval Period and the last 100 to 200 years, the warm status of the tropical Pacific Ocean forced the ITCZ to move northwards, which enhanced EASM intensity, but reduced ISM intensity. Meanwhile, the disproportional heating over the tropical Pacific Ocean may have led to decreased zonal SST gradients (analogous to a prolonged El Niño or El Niño-like status), which would have led to weakened Walker circulation, and weaker ISM intensity; and (2) During the LIA, the cold status of the tropical Pacific Ocean led to southern movement of the ITCZ, and intensified Walker circulation (analogous to a prolonged La Niña or La Niña-like status), resulting in weaker EASM and stronger ISM intensities, respectively.

## Methods

Proxy indices derived from lacustrine sedimentary archives along ETP areas, including Lakes Qinghai, Erhai, and Lugu (see Supplementary 1), were collected from our previous works (Supplementary 1). Optically Stimulated Luminescence (OSL) dating using materials from sediment core QH10-D was carried out in this study (Table S1) to test the reliability of the chronology of core QH10-A from Lake Qinghai (see Supplementary 1 for details).

In this study, we collected dateable materials, including aquatic snail remains buried in the paleo-shorelines/outcrops around Lake Chenghai, and determined their radiocarbon ages (Supplementary 1). To evaluate possible old carbon effects, we also collected modern lake water and living snails, and determined the corresponding ^14^C ages (see Supplementary 1 and Table S2). Historical lake levels were then determined based on the elevations of the paleo-shorelines/outcrops and the corresponding ages.

Sensitivity experiments were conducted to explore possible causes of the paleo-hydroclimatic contrasts inferred from the biogeological evidence mentioned in the text (see details in Supplementary 3).

Radiocarbon dating, OSL dating, and sensitivity experiments were conducted at the Institute of Earth Environment, Chinese Academy of Sciences (IEECAS).

## Additional Information

**How to cite this article**: Xu, H. *et al*. Hydroclimatic contrasts over Asian monsoon areas and linkages to tropical Pacific SSTs. *Sci. Rep.* **6**, 33177; doi: 10.1038/srep33177 (2016).

## Supplementary Material

Supplementary Information

## References

[b1] Jones P. D. & Mann M. E. Climate over past millennia. Rev. Geophys. 42, RG2002 (2004).

[b2] Diaz H. F. . Spatial and temporal characteristics of climate in medieval times revisited. Bull. Am. Meteorol. Soc. 92, 1487–1500 (2011).

[b3] Xu H. . Decadal/multi-decadal temperature discrepancies along the eastern margin of the Tibet plateau. Quat. Sci. Rev. 89, 85–93 (2014).

[b4] Chen J. H. . Hydroclimatic changes in China and surroundings during the Medieval Climate Anomaly and Little Ice Age: spatial patterns and possible mechanisms. Quat. Sci. Rev. 107, 98–111 (2015).

[b5] Zhang Q., Evans M. N. & Lyu L. Moisture dipole over the Tibetan Plateau during the past five and a half centuries. Nat. Commun. 6, doi: 10.1038/ncomms9062, (2015). PMC4560780 26293214

[b6] An Z. S. In Late Cenozoic Climate Change in Asia: Loess, Monsoon and Monsoon-arid Environment Evolution (Springer, 2014).

[b7] Xu H., Sheng E. G., Lan J. H., Liu B. & Yu K. K. Limnological records of the climatic changes along the eastern margin of the Tibetan Plateau during the past 2,000 years and their global linkages [in Chinese with English abstract]. Bull. Mineral. Petrol. Geochem. 34, 257–268 (2015a).

[b8] Yang B. . A 3,500-year tree-ring record of annual precipitation on the northeastern Tibetan Plateau. Proc. Natl. Acad. Sci. USA 111, 2903–2908 (2014). 24516152 10.1073/pnas.1319238111 PMC3939907

[b9] Zhang Q. B., Cheng G. D., Yao T. D., Kang X. C. & Huang J. G. A 2,326 year tree-ring record of climate variability on the northeastern Qinghai-Tibetan Plateau. Geophys. Res. Lett. 30, 1739–1742 (2003).

[b10] Shao X. M. . Reconstruction of precipitation variation from tree rings in recent 1000 years in Delingha, Qinghai. Sci. China Ser. D Earth Sci. 48, 939–949 (2005).

[b11] Tan L. C. . Climate patterns in north central China during the last 1800 yr and their possible driving force. Clim. Past 7, 685–692 (2011).

[b12] Xu H. . Late Holocene Indian summer monsoon variations recorded at Lake Erhai, Southwestern China. Quat. Res. 83, 307–314 (2015b).

[b13] Sheng E. G. . Late Holocene Indian summer monsoon precipitation history at Lake Lugu, northwestern Yunnan Province, southwestern China. Palaeogeogr. Palaeoclimatol. Palaeoecol. 438, 24–33 (2015).

[b14] Zhou Z. Y. & Zhao S. M. In The new compiling annals of Yunnan (Yunnan People’s Publishing House, 2007).

[b15] Chorography committee of Yongsheng County. In Chorography of ‘Yong Bei Zhi Li Ting Zhi’ (Yunnan University Press, 1999).

[b16] Xu H., Hong Y. T. & Hong B. Decreasing Asian summer monsoon intensity after 1860 AD in the global warming epoch. Clim. Dynam. 39, 2079–2088 (2012).

[b17] An Z. S. . Glacial-Interglacial Indian Summer Monsoon Dynamics. Science 333, 719–723 (2011). 21817044 10.1126/science.1203752

[b18] Ren G. Y. Pollen evidence for increased summer rainfall in the Medieval warm period at Maili, Northeast China. Geophys. Res. Lett. 25, 1931–1934 (1998).

[b19] Wu J. W., Lu R. J. & Zhao T. N. Sandy lands during the Medieval Warm Period in Eastern China [In Chinese with English abstract].Sci. Soil Water. Conserv. 2, 29–33 (2004).

[b20] Zhou Y. L. . Optically stimulated luminescence dating of aeolian sand in the Otindag dune field and Holocene climate change. Sci. China Ser. D Earth Sci. 51, 837–847 (2008).

[b21] Chen F. . East Asian summer monsoon precipitation variability since the last deglaciation. Scientific Reports 5, doi: 10.1038/srep11186 (2015). PMC4471663 26084560

[b22] Qin X. . Spectral analysis of a 1000-year stalagmite lamina-thickness record from Shihua Cave, Beijing, China. Holocene 9, 689–694 (1999).

[b23] Zhang D. E. Evidence for the existence of the Medieval Warm Period in China. Climatic Change 26, 289–297 (1994).

[b24] Ge Q. S., Zheng J. Y. & Fang X. Q. New understandings on the historical temperature changes in China [In Chinese with English abstract]. Prog. Geog. 21, 311–317 (2002).

[b25] Adhikari D. P. & Kumon F. Climatic changes during the past 1300 years as deduced from the sediments of Lake Nakatsuna, central Japan. Limnology 2, 157–168 (2001).

[b26] Yamada K. . Late Holocene monsoonal-climate change inferred from Lakes Ni-no-Megata and San-no-Megata, northeastern Japan. Quat. Int. 220, 122–132 (2010).

[b27] Kim G. S. & Choi I. S. In The Climate of China and Global Climate (eds Ye D. .) 30–37 (Springer, 1987).

[b28] Zhao Y., Yu Z. C. & Zhao W. W. Holocene vegetation and climate histories in the eastern Tibetan Plateau: controls by insolation-driven temperature or monsoon-derived precipitation changes? Quat. Sci. Rev. 30, 1173–1184 (2011).

[b29] Chu G. Q. . The ‘Mediaeval Warm Period’ drought recorded in Lake Huguangyan, tropical South China. Holocene 12, 511–516 (2002).

[b30] Tong G. B., Shi Y., Wu R. J., Yang X. D. & Qu W. C. Vegetation and climatic quantitative reconstruction of Longgan Lake since the past 3000 years [In Chinese with English abstract]. Mar. Geol. Quat. Geol. 17, 53–61 (1997).

[b31] He B., Zhang S. & Cai S. Climate changes recorded in peat from the Dajiu Lake basin in Shennongjia since the last 2600 years [In Chinese with English abstract]. Mar. Geol. Quat. Geol. 23, 109–115 (2003).

[b32] Sun L. G., Yan H. & Wang Y. H. South China Sea hydrological changes over the past millennium [in Chinese]. Chin. Sci. Bull. 57, 1730–1738 (2012).

[b33] Newton A., Thunell R. & Stott L. Climate and hydrographic variability in the Indo-Pacific Warm Pool during the last millennium. Geophys. Res. Lett. 33, L19710 (2006).

[b34] Tierney J. E., Oppo D. W., Rosenthal Y., Russell J. M. & Linsley B. K. Coordinated hydrological regimes in the Indo-Pacific region during the past two millennia. Paleoceanography 25, PA1102 (2010).

[b35] Wang B., Clemens S. C. & Liu P. Contrasting the Indian and East Asian monsoons: implications on geologic timescales. Mar. Geol. 201, 5–21 (2003).

[b36] Ding Y. & Chan J. C. L. The East Asian summer monsoon: an overview. Meteorol. Atmos. Phys. 89, 117–142 (2005).

[b37] Wang H. & Chen H. Climate control for southeastern China moisture and precipitation: Indian or East Asian monsoon? J. Geophys. Res. 117, D12109, doi: 10.1029/2012JD017734 (2012).

[b38] Verschuren D., Laird K. R. & Cumming B. F. Rainfall and drought in equatorial east Africa during the past 1,100 years. Nature 403, 410–414 (2000). 10667789 10.1038/35000179

[b39] Russell J. M. & Johnson T. C. A high-resolution geochemical record from Lake Edward, Uganda Congo and the timing and causes of tropical African drought during the late Holocene. Quat. Sci. Rev. 24, 1375–1389 (2005).

[b40] Stager J. C., Ryves D., Cumming B. F., Meeker L. D. & Beer J. Solar variability and the levels of Lake Victoria, East Africa, during the last millennium. J. Paleolimnol. 33, 243–251 (2005).

[b41] Mills K., Ryves D. B., Anderson N. J. & Bryant C. L. Expressions of climate perturbations in western Ugandan crater lake sediment records during the last 1000 years. Clim. Past 10, 1581–1601 (2014).

[b42] Thompson L. G. . Kilimanjaro Ice Core Records: Evidence of Holocene Climate Change in Tropical Africa. Science 298, 589–593 (2002). 12386332 10.1126/science.1073198

[b43] Miller C. S., Leroy S. A. G., Collins P. E. F. & Lahijani H. A. K. Late Holocene vegetation and ocean variability in the Gulf of Oman. Quat. Sci. Rev. 143, 120–132 (2016).

[b44] Zhang P. Z. . A test of climate, sun, and culture relationships from an 1810-year Chinese cave record. Science 322, 940–942 (2008). 18988851 10.1126/science.1163965

[b45] Hong B. . Abrupt variations of Indian and East Asian summer monsoons during the last deglacial stadial and interstadial. Quat. Sci. Rev. 97, 58–70 (2014).

[b46] Chao W. C. & Chen B. D. The origin of monsoons. J. Atmos. Sci. 58, 3497–3507 (2001).

[b47] Sachs J. P. . Southward movement of the Pacific Intertropical convergence zone AD 1400–1850. Nat. Geosci. 2, 519–525 (2009).

[b48] Kumar K. K., Rajagopalan B. & Cane M. A. On the weakening relationship between the Indian Monsoon and ENSO. Science 284, 2156–2159 (1999). 10381876 10.1126/science.284.5423.2156

[b49] Tokinaga H., Xie S. P., Deser C., Kosaka Y. & Okumura Y. M. Slowdown of the Walker circulation driven by tropical Indo-Pacific warming. Nature 491, 439–443 (2012). 23151588 10.1038/nature11576

[b50] Naidu C. V. . Is summer monsoon rainfall decreasing over India in the global warming era? J. Geophys. Res. 114, D24108 (2009).

[b51] Sinha A. . The leading mode of Indian Summer Monsoon precipitation variability during the last millennium. Geophys. Res. Lett. 38, L15703 (2011).

[b52] Tang S. & Qian W. Asian-Australian monsoon troughs and monsoon precipitation influenced by regional land-sea heating contrasts [In Chinese with English abstract]. J. Tropic. Meteorol. 25, 1–8 (2009).

[b53] Gupta S. K., Deshpande R. D., Bhattacharya S. K. & Jani R. A. Groundwater δ^18^O and δD from central Indian Peninsula: influence of the Arabian Sea and the Bay of Bengal branches of the summer monsoon. J. Hydrol. 303 38–55(2005)

[b54] Sengupta S. & Sarkar A. Stable isotope evidence of dual (Arabian Sea and Bay of Bengal) vapour sources in monsoonal precipitation over north India. Earth Planet. Sc. Lett. 250, 511–521 (2006).

[b55] Tan L. . Decreasing monsoon precipitation in southwest China during the last 240 years associated with the warming of tropical ocean. Clim Dyn., doi: 10.1007/s00382-016-3171-y (2016).

[b56] Prasad S. . Prolonged monsoon droughts and links to Indo-Pacific warm pool: A Holocene record from Lonar Lake, central India. Earth Planet. Sc. Lett. 391, 171–182 (2014).

[b57] Warrier A. K., Shankar R. & Sandeep K. Sedimentological and carbonate data evidence for lake level variations during the past 3700 years from a southern Indian lake. Palaeogeogr. Palaeoclimatol. Palaeoecol. 397, 52–60 (2014).

[b58] Cobb K. M., Charles C. D., Cheng H. & Edwards R. L. El Niño/Southern Oscillation and tropical Pacific climate during the last millennium. Nature 424, 271–276 (2003). 12867972 10.1038/nature01779

[b59] Oppo D. W., Rosenthal Y. & Linsley B. K. 2000-year-long temperature and hydrology reconstructions from the Indo-Pacific warm pool. Nature 460, 1113–1116 (2009). 19713927 10.1038/nature08233

[b60] Xie S. P. . Indian Ocean Capacitor Effect on Indo-Western Pacific Climate during the summer following El Niño. J. Clim. 22, 730–747 (2009).

[b61] Kalnay E. . The NCEP/NCAR 40-year reanalysis project. Bull. Am. Meteorol. Soc. 77, 437–470 (1996).

