# Supplementary material for: Hydroclimatic contrasts over Asian monsoon areas and linkages to tropical Pacific SSTs
Source: Sci Rep. 2016 Sep 9;6:33177. doi: 10.1038/srep33177 (PMC5016894; doi:10.1038/srep33177)
Supplement: Supplementary Information [file srep33177-s1.pdf]

Supplementary materials

Hydroclimatic contrasts over Asian monsoon areas and linkages to tropical Pacific SSTs

Hai Xu [1, 2], Jianghu Lan [1], Enguo Sheng [1], Bin Liu [1], Keke Yu [1], Yuanda Ye [1], Zhengguo Shi [1], Peng Cheng [1], Xulong Wang [1], Xinying Zhou [3], Kevin M. Yeager [4]

1. State key Laboratory of Loess and Quaternary Geology, institute of Earth Environment, Chinese Academy of Sciences. Xi'an, China.
2. Department of Environment Science and Technology, School of Human Settlements and Civil Engineering, Xi'an Jiaotong University, Xi'an, China.
3. Laboratory of Human Evolution and Archeological Science, Institute of Vertebrate Paleontology and Paleoanthropology, Chinese Academy of Sciences. Beijing, China.
4. Department of Earth and Environmental Sciences, University of Kentucky, Lexington, KY 40506, USA.

Correspondence should be addressed to Hai Xu: xuhai@ieecas.cn

Address:

Yanxiang Road, #97, Xi'an, Shaanxi province, China

Post Code: 710061

Tel: 86-29-62336295. Fax: 86-29-62336295

Mobile : 86-0-13991378151

Supplementary 1: Limnological evidence and data integration

Lake Qinghai, N-ETP

Sediment cores were collected at Lake Qinghai, northeastern Tibetan plateau in 2010. The activity of the radionuclide ^{137}Cs was measured in core QH10A (80 cm long), and the mass accumulation rate (MAR) was calculated [1]. Both the ^{137}Cs activity profile and MAR of core QH10A are similar to those of core #7 (collected about 150 m from core QH10A in 2007; *ref. 2*) (Fig. S1a). We then established an age model for core QH10A based on a constant sedimentation rate [1]. We also carried out ^{14}C dating for core QH10A (*ref. 1*; Table S1). However, our work showed that the ^{14}C ages of both plant debris and total organic carbon from core QH10A were too variable to generate a reliable chronology on decadal time scales. This is possibly a result of the erosion and transport of relatively old organic matter buried in the frozen soils within the catchment during the warm and wet seasons [1].

In this study, we determined Optically Stimulated Luminescence (OSL) ages of three samples from core QH10D (collected contemporaneously at the same site with core QH10A) using the single-aliquot regenerative-dose protocol with an additional annealing step [3]. The first two OSL ages match with the ^{137}Cs age model (Fig. S1b), while the third one (70-80 cm) does not. This suggested that the sedimentation rate below 70 cm may have been different from that above. We therefore only applied the ^{137}Cs age model to a maximum depth of 65 cm for core QH10A (time range from ~700 to 2010 AD).

Multi-proxy indices in core QH10A were determined, including sediment grain size, total organic carbon (TOC), C/N ratios of organic matter, and mass accumulation of organic carbon (MAoc) (Fig. S2), and the variations in these indices have been ascribed to changes in precipitation (*refs. 1, 4*; and see details in 'climatic significance of the proxy indices' in the section). As shown in Figures S2 and S3, these indices are well synchronized, and also match precipitation indicators from nearby, including tree ring widths [5, 6] and the drought/flood index [7, 8]. For example, the sedimentary grain size data indicate that droughts occurred at the intervals of ~870-940 AD, ~1090-1170 AD, ~1400-1520 AD, ~1640-1680 AD, ~1760-1830 AD, and ~1910-1940 AD at Lake Qinghai, which generally synchronize with those recorded in tree ring widths at Dulan and Delingha [7] (Fig. S3), supporting both the reliability of the age model of core QH10A and the robustness of the proxy indices. In this study, the grain size, C/N ratio, and MAoc data were used for comparison and/or data integration (Figs. S2, S3).

Lake Chenghai, S-ETP

Lake Chenghai is located in the northwestern Yunnan province, southeastern Tibetan plateau (Fig. 1). The modern lake is hydrologically closed, but lake water was discharged to the Jinsha River (upper reach of the Yangtze River) through the Chenghe River (the only one outlet of the

lake) several hundred years ago [9]. The elevation of the dividing point of the Chenghe River bed is about 43 m above the modern lake level (Alt. ~1500 m), and today the river is dry. Changes in paleo-lake levels are important in the context of local hydrological variations, and are therefore crucial to understanding the variations in ISM intensity.

Relative lake levels can be derived from historical literature. For example, historical literature recorded that the lake level was high and Lake Chenghai was termed a "sea" during the Yuan Dynasty (1271 to 1368 AD; *ref. 10*). Lake level began to decrease during the middle Ming Dynasty (1368 to 1644 AD), and people constructed a dam (*Elevation: ~ 1543 m, estimated from its historical location; this study*) across the Chenghe River to store water during the Wanli Empire (1573-1620 AD) [10]. The dam was repeatedly rebuilt, and the river channel was widened several times during the Qing Dynasty (1636 to 1912 AD) due to lake level fluctuations as recorded in *New Yunnan chorography* [10]. These historical records suggest that lake levels were considerably higher during the Little Ice Age (LIA) (close to the elevation of the dividing point of the Chenghe River bed, ~1543 m), because if not, there would have been no need for dam construction. The lake level sharply dropped by ~33 meters in ~1779 AD as recorded in the chorography of *Yong Bei Zhi Li Ting Zhi* [11]. Thereafter, lake levels remained low and the dam was abandoned [1].

Former lake levels can also be physically reconstructed using the ages and elevations of paleo-shorelines/terraces. In this study, we investigated paleo-shorelines/terraces around Lake Chenghai (Fig. S4), and collected materials suitable for ^{14}C dating (e.g., snail shells, Table S2). We also determined the ^{14}C ages of living snails and lake water samples to evaluate a possible old carbon effect. As shown in Table S2, the ^{14}C pMC values of living snails and lake water samples suggest that there is no obvious old carbon effect in Lake Chenghai, and therefore fossil snail ages from paleo-shorelines/terraces accurately record the ages of the historical lake levels. As shown in Figure S4 and Table S2, low lake levels frequently occurred during the Medieval Period as compared with the high stands during LIA, faithfully indicating ISM weakenings during this time.

Lake Erhai, S-ETP

Lake Erhai is located approximately 80 km to the southwest of Lake Chenghai (Fig. 1). We previously collected sediment cores from Lake Erhai and established an age model by combining ^{137}Cs, ^{210}Pb, and ^{14}C ages [12]. Both the ^{137}Cs and ^{210}Pb age models are similar to those from previous work at Lake Erhai [12]. However, the ^{14}C ages used in our study differed from those from some previous work by approximately 520-610 years. We attribute this difference to the old carbon effect, as our data were corrected for this effect, while those data from previous work were not [12]. The age model for the last one hundred years has been verified using Eucalyptus-pollen, as Eucalyptus trees in the Lake Erhai catchment are known to have been introduced about one hundred years ago [12]. Multi-proxy indices were applied to study climatic changes at Lake Erhai over the late Holocene, and conifer pollen concentrations (Fig. 3) was used as an indicator of

precipitation in this study [12].

Lake Lugu, S-ETP

Lake Lugu is also located in the northwestern Yunnan province, southeastern Tibetan plateau (Fig. 1). We collected sediment cores from the lake and established age models based on ^{14}C ages of plant debris [13]. Changes in lake sediment grain size are primarily controlled by changes in monsoon precipitation intensity here. Changes in TOC and C/N ratios in lake sediments are also mainly ascribed to changes in ISM intensity (*refs.13*; see details in 'climatic significance of the proxy indices' in this section). In this study, sediment grain size and C/N ratios (Fig. 3) were used to indicate trends of precipitation during the past 2,000 years here.

Other data collection and integration

Evidence from other published works was collected for comparison (Table S3). We standardized proxy indices over the S-ETP and N-ETP areas, and made 10-yr interpolations. Stacked S-ETP and N-ETP time series were then generated by simply averaging the standardized proxy indices (Figs. S2, S3). In this study, the synthesized precipitation curve over S-ETP areas was generated using conifer pollen concentration (%) data from Lake Erhai [12], and sediment grain size and C/N ratio data from Lake Lugu [13]; while that over N-ETP areas was derived from sediment grain size, C/N ratios, and MA_{oc} data from Lake Qinghai, tree ring records at Delingha [6], Dulan [5], and Qilian Mt. [14], and the drought/flood index from the Longxi area [7, 8] (see locations of the sites in Fig. 1).

Climatic significance of the proxy indices

Generally, the total organic carbon (TOC) content reflects the biomass both in the lake and the catchment. Terrestrial plants and/or emergent plants are rich in fiber, but poor in protein, and the atomic C/N ratios of organic matter are therefore high (generally greater than 20; *refs. 4, 12*). In contrast, lake algae and/or plankton contain less fiber, but more protein, and hence have low atomic C/N ratios (generally less than 10; *refs. 4, 12*). As a result, the atomic C/N ratios of organic matter have been widely used to indicate the relative contribution of authigenic and terrigenous organic matter. Higher C/N ratio values correlate to larger proportions of terrigenous organic matter; while lower C/N ratio values imply higher proportions of algal organic matter. The aquatic plants will partly use the dissolved inorganic carbon (DIC) during photosynthesis, usually leading to higher $\delta^{13}C_{org}$ of algal and/or plankton than that of C_3 plants in catchments [4, 12]. Therefore, lower $\delta^{13}C$ of total organic matter indicates higher contribution of terrestrial organic matter, while higher $\delta^{13}C$ of total organic matter indicates higher contribution of algal organic matter [4, 12]. In addition, increased rainfall leads to increased surface runoff, bringing larger terrestrial particles to the lake and leading to a greater grain size of the sediment in the center of the lake, and vice versa [13]. As a result, the sedimentary TOC, C/N ratio, $\delta^{13}C_{org}$, and grain size are widely used as indicators to trace changes in precipitation across the ETP areas (e.g., *refs. 1, 12, 13*).

The climatic significance of the pollen data may be variable on different spatial and temporal scales. In the Lake Erhai catchment, because *Abies* and *Picea* favor shaded, cool and wet environments, and are sensitive to strong droughts, and because the fir and pine trees around Lake Erhai are selectively distributed within a cool elevation zone (between 2,500~3,500 m), fluctuations in their abundances on short term time scales (like decadal/multi-decadal timescales) are most likely to be primarily controlled by variations in precipitation. Therefore, the decadal/multi-decadal time scale variations in conifer pollen concentrations in Lake Erhai sediment was used as an indicator of precipitation (see *refs. 12* for details).

Supplementary 2: Water vapor sources of the ETP areas.

Water vapor over the southern ETP (S-ETP) is controlled by ISM precipitation, as has been shown by several previous studies (e.g., *refs. 15-18*). Water vapor over the northern ETP (N-ETP) may be supplied by both the EASM and the westerly jet stream (e.g., *ref. 19*). It is interesting to note that the Medieval wet climate over N-ETP areas (750~1200 AD; e.g., *refs. 1, 5, 14*) clearly occurred earlier than in Europe (900 to 1350 AD; e.g., *refs. 20, 21*), which possibly implies that the westerly jet stream may not have played a leading role in transporting water vapor to N-ETP areas during the Medieval Period. Xu et al. [22] showed a close correlation between sea surface temperatures (SST) in the eastern tropical Pacific Ocean ($SST_{Niño3}$) and the drought/flood index in Xining, suggesting that precipitation over N-ETP areas is closely related to the EASM over multi-annual to decadal time scales. It is likely that summer water vapor over the N-ETP was also controlled by changes in EASM intensity during the past 2,000 years.

Supplementary 3: Sensitivity experiments

Numerical experiments were performed to evaluate the responses of Asian summer monsoon precipitation to the surface warming and/or cooling of the tropical Pacific Ocean. The model used in this study is the Community Atmosphere Model version 3 (CAM3) [23], an atmospheric general circulation model developed by the National Center for Atmospheric Research (NCAR). CAM3 is the atmospheric component of the Community Climate System Model version 3 (CCSM3) and has been widely employed to simulate past, present and future climate changes. In CAM3, the Community Land Model version 3 (CLM3) is coupled to calculate land surface processes. For the control experiment, all boundary conditions, including ice cover, vegetation, and SST, are fixed at contemporary values. The SST field for the model is merged from the HadISST/Reynolds data set [24]. The concentrations of greenhouse gases are set to pre-industrial values (CO_2 = 280 ppm, CH_4 = 700 ppb, N_2O = 275 ppb). To examine model sensitivity to tropical Pacific warming, we kept all other conditions the same but simply modified the SST fields. Both warming and cooling scenarios (compared with modern SST values) were applied over the eastern (210-270°E, 5°S-5°N), central (160-210°E, 5°S-5°N), western (100-160°E, 5°S-5°N), and the entire (100-270°E, 5°S-5°N) tropical Pacific Oceans. All experiments were performed at a horizontal resolution of T42, which corresponds approximately to 2.8° × 2.8°. After a spin-up time of 10 years, each experiment was integrated for another 40 years and the corresponding results were averaged for analyses. The results of the sensitivity experiments for the entire tropical Pacific Ocean are shown in Figure 4 (in the main text), while those for the eastern, central, and western tropical Pacific Ocean are provided in Figure S5.

Supplementary 4: Possible Medieval SST over tropical Pacific Ocean

Variations in SSTs in the eastern tropical Pacific Ocean are critical to understanding global climate dynamics because they are closely related to the north-south movement of the Intertropical Convergence Zone (ITCZ) and the intensification/weakening of Walker circulation. However, SST variations over the eastern tropical Pacific Ocean are poorly known. Cobb et al. [25] reconstructed SSTs in this region over the past ~1,000 years using coral $\delta^{18}O$ data and their results indicated colder SSTs during the Medieval Period than during the Little Ice Age. However, the comparisons of the reconstructed SSTs between the Medieval Period and LIA may be problematic, as the corals are collected from different sites with different micro-environments, such as water depth, temperature, and sea water $\delta^{18}O$ values. In addition, the coral numbers during the Medieval Period are also limited as compared with those during other time intervals [25]. Conroy et al. [26] resolved high-resolution climatic changes at Lake El Junco, a small lake on the island of San Cristóbal, in the Galápagos Islands, and the results suggest a much warmer Medieval Period with a high frequency of El Niño events (Fig. S6). Speleothem $\delta^{18}O$ records from the Isthmus of Panama also suggest much higher El Niño frequency during the Medieval Period [27]. Modern observations show that precipitation over Ecuador and northern Peru has increased significantly during warmer stages (El Niño) in the tropical eastern Pacific Ocean (e.g., *refs. 28-32*). Much stronger terrestrial runoff (wetter conditions) can be inferred from the sedimentation rate at Lake Laguna Pallcacocha in Ecuador during the Medieval Period, which supports an El Niño or El Niño-like status during this interval (Fig. S6; *refs. 32, 33*). Pollen records from a bog in the eastern Ecuadorian Andes indicated warm and moist climatic conditions during the Medieval Period, suggesting higher ENSO variability between 850 and 1250 AD [34]. These lines of evidence indicate a scenario of warmer SSTs over the tropical eastern Pacific Ocean during the Medieval times.

References

1. Xu, H., Sheng, E. G., Lan, J. H., Liu, B. & Yu, K. K. Limnological records of the climatic changes along the eastern margin of the Tibetan Plateau during the past 2,000 years and their global linkages [in Chinese with English abstract]. *Bull. Mineral. Petrol. Geochem.* **34,** 257-268 (2015).
2. Xu, H. *et al.* Spatial pattern of modern sedimentation rate of Qinghai Lake and a preliminary estimate of the sediment flux. *Chin. Sci. Bull.* **55,** 621-627 (2010).
3. Du, J. & Wang, X. Optically stimulated luminescence dating of sand-dune formed within the Little Ice Age. *J. Asian Earth Sci.* **91,** 154-162 (2014).
4. Xu, H., Ai, L., Tan, L. C. & An, Z. S. Stable isotopes in bulk carbonates and organic matter in recent sediments of Lake Qinghai and their climatic implications. *Chem. Geol.* **235,** 262-275 (2006).
5. Zhang, Q. B., Cheng, G. D., Yao, T. D., Kang, X. C. & Huang, J. G. A 2,326 year tree-ring record of climate variability on the northeastern Qinghai-Tibetan Plateau. *Geophys. Res. Lett.* **30,** 1739-1742 (2003).
6. Shao, X. M. *et al.* Reconstruction of precipitation variation from tree rings in recent 1000 years in Delingha, Qinghai. *Sci. China Ser. D Earth Sci.* **48,** 939-949 (2005).
7. Tan, L. C., Cai, Y. J., Yi, L., An, Z. S. & Ai, L. Precipitation variations of Longxi, northeast margin of Tibetan Plateau since AD 960 and their relationship to solar variability. *Clim. Past* **4,** 19-28 (2008).
8. Tan, L. C. *et al.* Climate patterns in north central China during the last 1800 yr and their possible driving force. *Clim. Past* **7,** 685-692 (2011).
9. Wang, S. M. & Dou, H. S. in *Lakes in China [in Chinese]* (Academic Publisher, 1998).
10. Zhou, Z. Y. *et al.* in *The new compiling annals of Yunnan* (Yunnan People's Publishing House, 2007).
11. Chorography committee of Yongsheng County. in *Chorography of 'Yong Bei Zhi Li Ting Zhi'* (Yunnan University Press, 1999).
12. Xu, H. *et al.* Late Holocene Indian summer monsoon variations recorded at Lake Erhai, Southwestern China. *Quat. Res.* **83,** 307-314 (2015).
13. Sheng, E. G. *et al.* Late Holocene Indian summer monsoon precipitation history at Lake Lugu, northwestern Yunnan Province, southwestern China. *Palaeogeogr. Palaeoclimatol. Palaeoecol.* **438,** 24-33 (2015).
14. Yang, B. *et al.* A 3,500-year tree-ring record of annual precipitation on the northeastern Tibetan Plateau. *Proc. Natl. Acad. Sci. USA* **111,** 2903-2908 (2014).
15. Hong, Y. T. *et al.* Correlation between Indian Ocean summer monsoon and North Atlantic climate during the Holocene. *Earth Planet. Sci. Lett.* **211,** 371-380 (2003).
16. An, Z. S. *et al.* Glacial-Interglacial Indian Summer Monsoon Dynamics. *Science* **333,** 719-723 (2011).
17. Xu, H., Hong, Y. T. & Hong, B. Decreasing Asian summer monsoon intensity after 1860 AD in the global warming epoch. *Clim. Dynam.* **39,** 2079-2088 (2012).
18. Sano, M. *et al.* May-September precipitation in the Bhutan Himalaya since 1743 as reconstructed from tree ring cellulose $\delta^{18}O$. *J. Geophys. Res.* **118,** 8399-8410 (2013).
19. An, Z. S. *et al.* Interplay between the Westerlies and Asian monsoon recorded in Lake Qinghai sediments since 32 ka. *Sci. Rep.* **2,** 619 (2012).
20. Graham, N. E., Ammann, C. M., Fleitmann, D., Cobb, K. M. & Luterbacher, J. Support for global climate reorganization during the "Medieval Climate Anomaly". *Clim. Dynam.* **37,**

1217-1245 (2011).

21. Diaz, H. F. *et al.* Spatial and temporal characteristics of climate in medieval times revisited. *Bull. Am. Meteorol. Soc.* **92,** 1487-1500 (2011).

22. Xu, H., Hou, Z. H., Ai, L. & Tan, L. C. Precipitation at Lake Qinghai and its relation to Asian summer monsoons on decadal/interdecadal scales during the past 500 years. *Palaeogeogr. Palaeoclimatol. Palaeoecol.* **254,** 541-549 (2007).

23. Collins, W. D. *et al.* The formulation and atmospheric simulation of the Community Atmosphere Model Version 3 (CAM3). *J. Clim.* **19,** 2144-2161 (2006).

24. Hurrell, J. W., Hack, J. J., Shea, D., Caron, J. M. & Rosinski, J. A new sea surface temperature and sea ice boundary dataset for the Community Atmosphere Model. *J. Clim.* **21,** 5145-5153 (2008).

25. Cobb, K. M., Charles, C. D., Cheng, H. & Edwards, R. L. El Niño/Southern Oscillation and tropical Pacific climate during the last millennium. *Nature* **424,** 271-276 (2003).

26. Conroy, J. L. *et al.* Unprecedented recent warming of surface temperatures in the eastern tropical Pacific Ocean. *Nature Geosci.* **2,** 46-50 (2009).

27. Lachniet, M. S. *et al.* A 1500-year El Niño/Southern Oscillation and rainfall history for the Isthmus of Panama from speleothem calcite. *J. Geophys. Res.* **109,** D20117 (2004).

28. Tapley, T. D. & Waylen, P. R. Spatial variability of annual precipitation and ENSO events in western Peru. *J. Hydrol. Sci.* **35,** 429-446 (1990).

29. Coelho, C. A. S., Uvo, C. B. & Ambrizzi, T. Exploring the impacts of the tropical Pacific SST on the precipitation patterns over South America during ENSO periods. *Theor. Appl. Climatol.* **71,** 185-197 (2002).

30. Haylock, M. R. *et al.* Trends in total and extreme South American rainfall in 1960-2000 and links with sea surface temperature. *J. Clim.* **19,** 1490-1512 (2006).

31. Rodbell, D. T. *et al.* An ~15,000-year record of El Niño-driven alluviation in southwestern Ecuador. *Science* **283,** 516-520 (1999).

32. Moy, C. M., Seltzer, G. O., Rodbell, D. T. & Anderson, D. M. Variability of El Niño/Southern Oscillation activity at millennial timescales during the Holocene epoch. *Nature* **420,** 162-165 (2002).

33. Tierney, J. E., Oppo, D. W., Rosenthal, Y., Russell, J. M. & Linsley, B. K. Coordinated hydrological regimes in the Indo-Pacific region during the past two millennia. *Paleoceanography* **25,** PA1102 (2010).

34. Ledru, M. P. *et al.* The Medieval Climate Anomaly and the Little Ice Age in the eastern Ecuadorian Andes. *Clim. Past* **9,** 307-321 (2013).

35. Chu, G. Q. *et al.* The 'Mediaeval Warm Period' drought recorded in Lake Huguangyan, tropical South China. *Holocene* **12,** 511-516 (2002).

36. He, B., Zhang, S. & Cai, S. Climate changes recorded in peat from the Dajiu Lake basin in Shennongjia since the last 2600 years [In Chinese with English abstract]. *Mar. Geol. Quat. Geol.* **23,** 109-115 (2003).

37. Oppo, D. W., Rosenthal, Y. & Linsley, B. K. 2000-year-long temperature and hydrology reconstructions from the Indo-Pacific warm pool. *Nature* **460,** 1113-1116 (2009).

38. Stuiver, M. *et al.* INTCAL98 radiocarbon calibration, 24,000-0 cal BP. *Radiocarbon* **40,** 1041-1083 (1998).

39. Shen, J., Liu, X. Q., Wang, S. M. & Matsumoto, R. Palaeoclimatic changes in the Qinghai Lake area during the last 18,000 years. *Quat. Int.* **136,** 131-140 (2005).

40. Halfman, J. D., Johnson, T. C. & Finney, B. New AMS dates, stratigraphic correlations and decadal climate cycles for the past 4 ka at Lake Turkana, Kenya. *Palaeogeogr. Palaeoclimatol.*

Palaeoecol. **111,** 83-98 (1994).

41. Russell, J. M. & Johnson, T. C. A high-resolution geochemical record from Lake Edward, Uganda Congo and the timing and causes of tropical African drought during the late Holocene. *Quat. Sci. Rev.* **24,** 1375-1389 (2005).
42. Stager, J. C., Ryves, D., Cumming, B. F., Meeker, L. D. & Beer, J. Solar variability and the levels of Lake Victoria, East Africa, during the last millennium. *J. Paleolimnol.* **33,** 243-251 (2005).
43. Mills, K., Ryves, D. B., Anderson, N. J. & Bryant, C. L. Expressions of climate perturbations in western Ugandan crater lake sediment records during the last 1000 years. *Clim. Past* **10,** 1581-1601 (2014).
44. Verschuren, D., Laird, K. R. & Cumming, B. F. Rainfall and drought in equatorial east Africa during the past 1,100 years. *Nature* **403,** 410-414 (2000).
45. Thompson, L. G. *et al.* Kilimanjaro Ice Core Records: Evidence of Holocene Climate Change in Tropical Africa. *Science* **298,** 589-593 (2002).
46. Fleitmann, D. *et al*. Holocene Forcing of the Indian Monsoon Recorded in a Stalagmite fromSouthern Oman. *Science* **300**, 1737-1739 (2003).
47. Miller, C.S., Leroy, S.A.G., Collins, P.E.F. & Lahijani, H. A.K. Late Holocene vegetation and ocean variability in the Gulf of Oman. *Quat. Sci. Rev*. **143**,120-132 (2016).
48. Prasad, S. et al. Prolonged monsoon droughts and links to Indo-Pacific warm pool: A Holocene record from Lonar Lake, central India. *Earth Planet. Sc. Lett.* **391**,171-182 (2014).
49. Warrier, A. K., Shankar, R. & Sandeep, K. Sedimentological and carbonate data evidence for lake level variations during the past 3700 years from a southern Indian lake. *Palaeogeogr. Palaeoclimatol. Palaeoecol.* **397**, 52-60 (2014).
50. Newton, A., Thunell, R. & Stott, L. Climate and hydrographic variability in the Indo-Pacific Warm Pool during the last millennium. *Geophys. Res. Lett.* **33,** L19710 (2006).
51. Sun, L. G., Yan, H. & Wang, Y. H. South China Sea hydrological changes over the past millennium [in Chinese]. *Chin. Sci. Bull.* **57,** 1730-1738 (2012).
52. Tong, G. B., Shi, Y., Wu, R. J., Yang, X. D. & Qu, W. C. Vegetation and climatic quantitative reconstruction of Longgan Lake since the past 3000 years [In Chinese with English abstract]. *Mar. Geol. Quat. Geol.* **17,** 53-61 (1997).
53. Zhang, P. Z. *et al.* A test of climate, sun, and culture relationships from an 1810-year Chinese cave record. *Science* **322,** 940-942 (2008).
54. Wu, J. W., Lu, R. J. & Zhao, T. N. Sandy lands during the Medieval Warm Period in Eastern China [In Chinese with English abstract]. *Sci. Soil Water. Conserv.* **2,** 29-33 (2004).
55. Chen, F. *et al*. East Asian summer monsoon precipitation variability since the last deglaciation. *Scientific Reports* **5**, doi: 10.1038/srep11186 (2015)..
56. Qin, X. *et al.* Spectral analysis of a 1000-year stalagmite lamina-thickness record from Shihua Cave, Beijing, China. *Holocene* **9,** 689-694 (1999).
57. Zhou, Y. L. *et al.* Optically stimulated luminescence dating of aeolian sand in the Otindag dune field and Holocene climate change. *Sci. China Ser. D Earth Sci.* **51,** 837-847 (2008).
58. Ren, G. Y. Pollen evidence for increased summer rainfall in the Medieval warm period at Maili, Northeast China. *Geophys. Res. Lett.* **25,** 1931-1934 (1998).
59. Kim, G. S. & Choi, I. S. in *The Climate of China and Global Climate* (eds Ye, D. *et al.*) 30-37 (Springer, 1987).
60. Adhikari, D. P. & Kumon, F. Climatic changes during the past 1300 years as deduced from the sediments of Lake Nakatsuna, central Japan. *Limnology* **2,** 157-168 (2001).
61. Yamada, K. *et al.* Late Holocene monsoonal-climate change inferred from Lakes

Ni-no-Megata and San-no-Megata, northeastern Japan. *Quat. Int.* **220,** 122-132 (2010).

62. Sinha A, Kathayat G, Cheng H, Breitenbach SFM, Berkelhammer M, Mudelsee M*, et al.* Trends and oscillations in the Indian summer monsoon rainfall over the last two millennia. *Nat Commun* 2015, **6**.

63. Sanwal, J., Kotlia, B. S., Rajendran,C., Ahmad, S.M., Rajendran, K. &Sandiford, M. Climatic variability in Central Indian Himalaya during the last ~1800 years: Evidence from a high resolution speleothem record. Quat. Int. 304, 183-192 (2013).

64. Sinha, A. *et al.* The leading mode of Indian Summer Monsoon precipitation variability during the last millennium. *Geophys. Res. Lett.* **38,** L15703 (2011).

65. Sinha A, Cannariato KG, Stott LD, Cheng H, Edwards RL, Yadava MG*, et al.* A 900-year (600 to 1500 A. D.) record of the Indian summer monsoon precipitation from the core monsoon zone of India. *Geophys Res Lett* 2007, **34**(16).

Figure Captions and Table Titles

Figure S1. a (left panel). ^{137}Cs radioactivities of samples in core QH10A (blue triangle line; *ref. 1*) and in core #7 (pink circle line; *ref. 2*). Blue dotted line shows a manual fit of the ^{137}Cs peak for core QH10A. b (right panel). ^{137}Cs age model of core QH10A and the ^{14}C ages of bulk organic matter (yellow cross- square; *ref. 1*), and the OSL ages (blue triangle; this study) for core QH 10-D (Table S1). Note the ^{137}Cs age model was applied from 0 to 65cm (blue diamonds) while that below (65-85cm; red diamonds) was discarded.

Figure S2. Proxy indices in core QH10A at Lake Qinghai during the past 1,300 years (Redrawn from *ref. 1*). a. total organic carbon content (TOC; red), b. δ^{13}C of organic matter (pink), c. mass accumulation of organic carbon (MA_{oc}; blue), d. C/N ratio (green), and e. grainsize (purple). Also shown is the stacked precipitation index in core QH0407C (f; orange) developed by our previous work [4].

Figure S3. Comparison between precipitation indices of Lake Qinghai and those nearby during the past 1,300 years. a. Qilian Mt. precipitation [14]. b. Delingha precipitation reconstructed from tree ring widths (orange; normalized) [6].c. Dulan tree ring width index (precipitation indicator; blue) [5], d. grainsize of core QH10A (purple) [1]. e. C/N ratio values of core QH10A (green) [1], and f. the drought/flood (D/F) index at Longxi (pink) [7, 8]. The grey shaded column highlights the medieval period over the N-ETP. The yellow shaded columns show synchronicities of the decadal/multi-decadal climatic changes over N-ETP areas.

Figure S4. Outcrops/shorelines showing low lake levels (as compared with the dividing height of Lake Chenghai: ~1543m) during the medieval period. See dating results in Table S2.

CHP5-3: Alt. ~1520 m. This outcrop was cut out by river. Laminated layers are clear and plenty small snail remains are buried in different layers of the profile.

CHP5-2: Alt. ~1512 m. This outcrop is located roadside, and small snail remains were found.

CHP3-1: Alt. ~1507 m. This outcrop/shoreline located aside a riverbed, and snail remains and a piece of ceramic debris ("whiteware") were found within the profile.

CHP6-2: Alt. ~1512 m. This paleao-shoreline was located roadside, and plenty of small snail remains were found.

Figure S5. Responses of monsoon precipitation over EASM and ISM areas to changes in SST over tropical Pacific Ocean. A and B show the results of the 1℃ warming and cooling sensitive experiments over the central Pacific Ocean, respectively. 'C and D', 'E and F' show the results of similar experiments except that the experiment regions are eastern- and western- tropical Pacific, respectively. The legend shows changes in precipitation (mm/d). The sites are similar to those in Figure 1 (in the main text) except that all of the sites in ISM areas are changed to green (for a better color contrast). Dotted areas represent significant levels higher than 95%. The simulations and the basemaps were drawn in Grid Analysis and Display System (GrADS) 1.9.

Figure S6. Comparison between hydroclimatic changes over south China and the El Nino frequency over eastern tropical Pacific region. a (green), Lake Lugu C/N ratios [13]. b (pink), Lake Huguangyan C/N ratios [35]. c (blue), precipitation reconstructed from pollen records at Lake Dajiuhu [36]. d. SST over the Western Pacific Warm Pool areas (WPWP) [37]. e (purple), sand% at El Junco, eastern tropical Pacific (higher value corresponds to higher El Niño frequency; *ref. 26*). f (grey), red intensity index of lake sediments in Lake Laguna Pallcacocha (higher value corresponds to higher El Niño frequency; *ref. 32*). The yellow shaded columns sketchily show the medieval period and the last 100-200 years.

Table S.1. ^{14}C ages and OSL ages for samples at Lake Qinghai

Table S.2. ^{14}C ages of the snail remains in the paleao-shorelines/profiles, and those of the living snails and modern lake waters at Lake Chenghai

Table S.3. Sites mentioned in this study: the ISM region (1-20), EASM region (21-38), and the northern India (39-43).

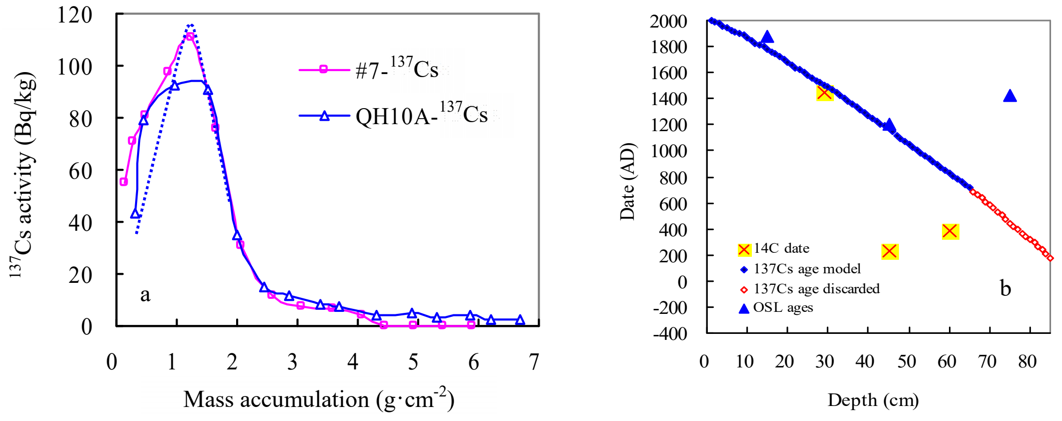

120
100
80
60
40
20
0
^{137}Cs activity (Bq/kg)
#7-^{137}Cs
QH10A-^{137}Cs
a
0 1 2 3 4 5 6 7
Mass accumulation (g·cm^{-2})
2000
1800
1600
1400
1200
1000
800
600
400
200
0
-200
-400
Date (AD)
14C date
137Cs age model
137Cs age discarded
OSL ages
b
0 10 20 30 40 50 60 70 80
Depth (cm)

Figure S1.

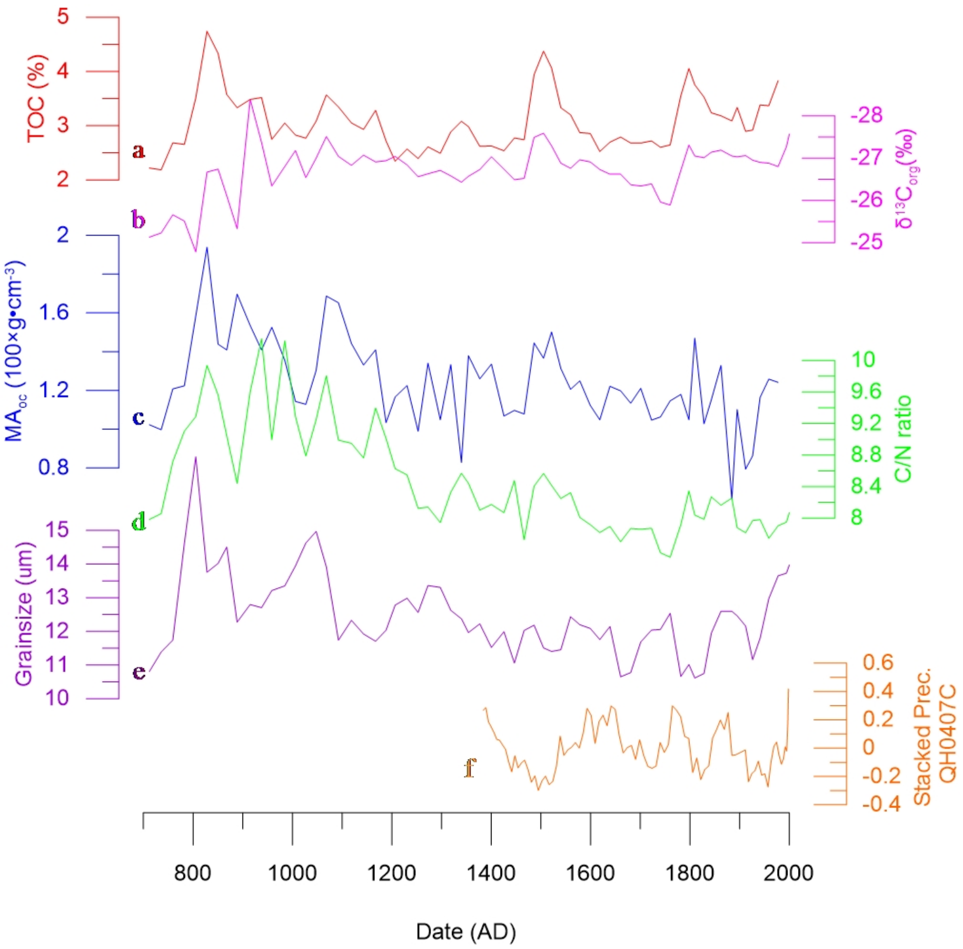

TOC (%)
5
4
3
2
a
δ13Corg(‰)
-28
-27
-26
-25
b
MAoc (100×g•cm-3)
2
1.6
1.2
0.8
c
C/N ratio
10
9.6
9.2
8.8
8.4
8
d
Grainsize (um)
15
14
13
12
11
10
e
Stacked Prec. QH0407C
0.6
0.4
0.2
0
-0.2
-0.4
f
800
1000
1200
1400
1600
1800
2000
Date (AD)

Figure S2.

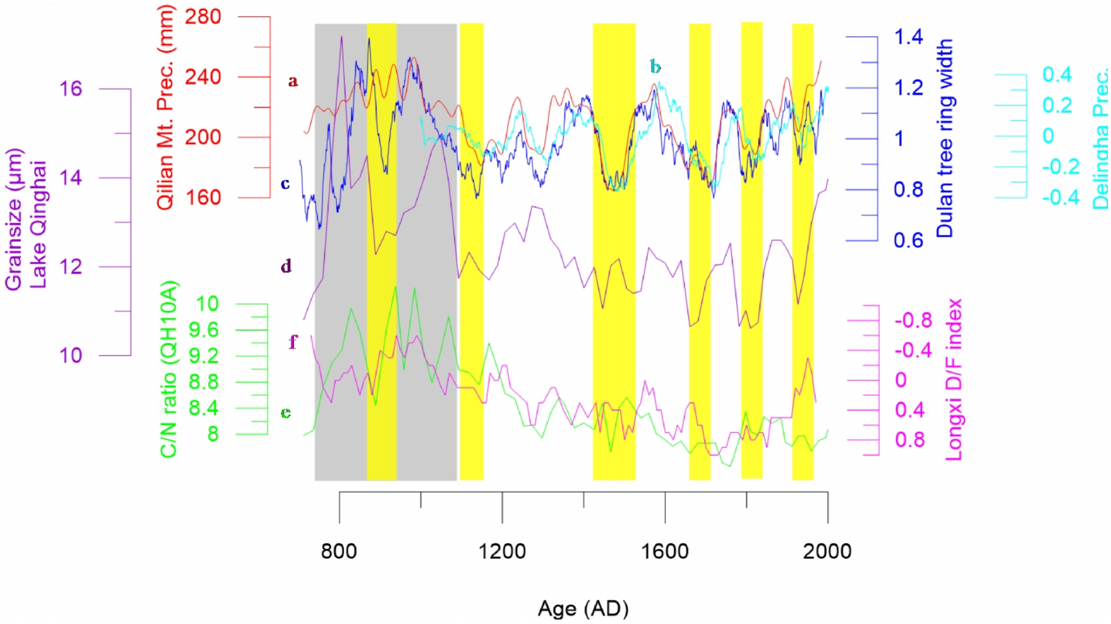
Grainsize (μm)
Lake Qinghai
16
14
12
10
Qilian Mt. Prec. (mm)
280
240
200
160
C/N ratio (QH10A)
10
9.6
9.2
8.8
8.4
8
a
b
c
d
f
e
1.4
1.2
1
0.8
0.6
Dulan tree ring width
0.4
0.2
0
-0.2
-0.4
Delingha Prec.
-0.8
-0.4
0
0.4
0.8
Longxi D/F index
800
1200
1600
2000
Age (AD)

Figure S3.

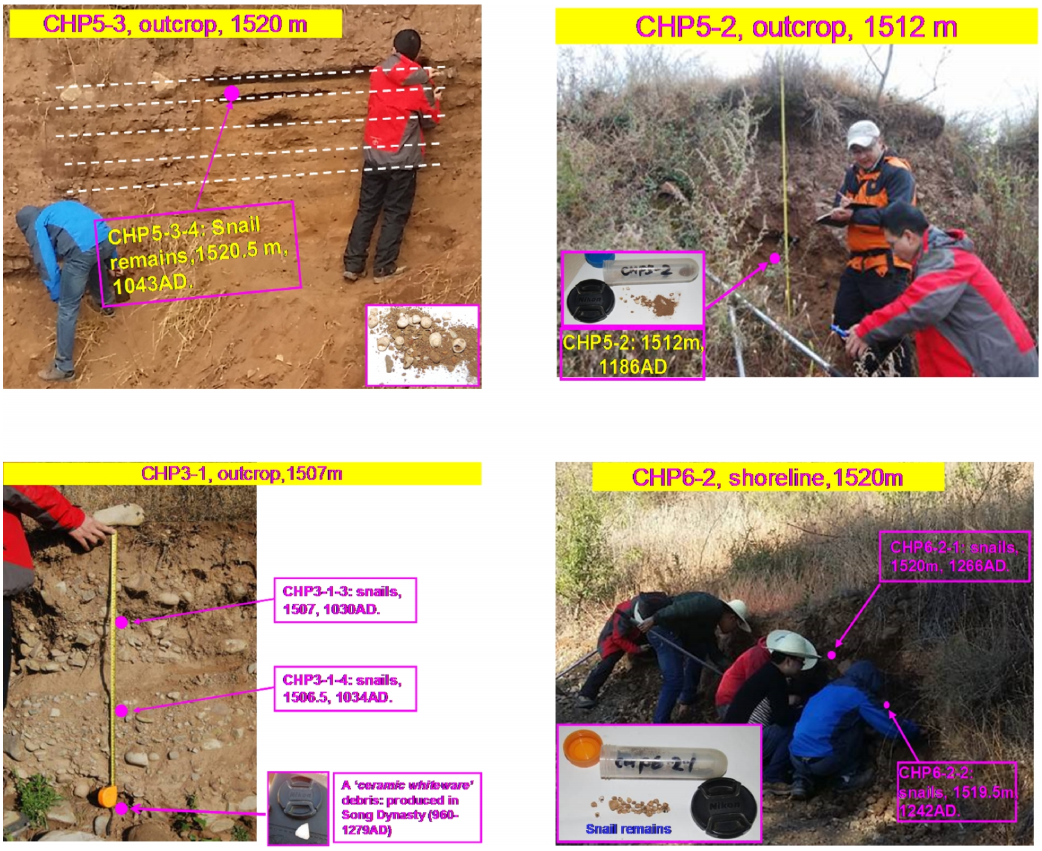
CHP5-3, outcrop, 1520 m
CHP5-3-4: Snail remains,1520.5 m, 1043AD.
CHP5-2, outcrop, 1512 m
CHP5-2: 1512m, 1186AD
CHP3-1, outcrop,1507m
CHP3-1-3: snails, 1507, 1030AD.
CHP3-1-4: snails, 1506.5, 1034AD.
A 'ceramic whiteware' debris: produced in Song Dynasty (960-1279AD)
CHP6-2, shoreline,1520m
CHP6-2-1: snails, 1520m, 1266AD.
CHP6-2-2: snails, 1519.5m, 1242AD.
Snail remains

Figure S4.

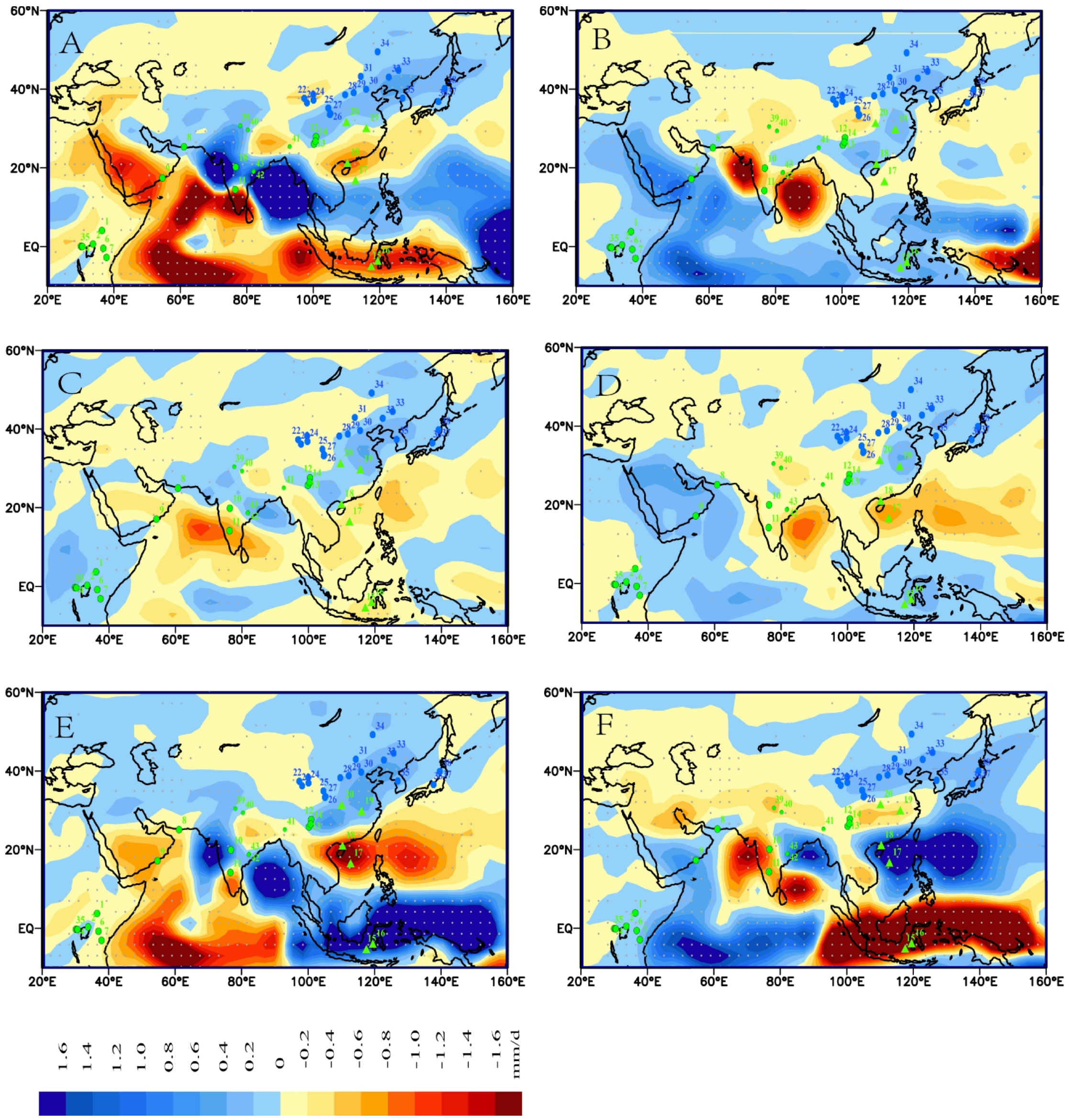
A
B
C
D
E
F
60°N
40°N
20°N
EQ
20°E
40°E
60°E
80°E
100°E
120°E
140°E
160°E
1.6
1.4
1.2
1.0
0.8
0.6
0.4
0.2
0
-0.2
-0.4
-0.6
-0.8
-1.0
-1.2
-1.4
-1.6
mm/d

Figure S5.

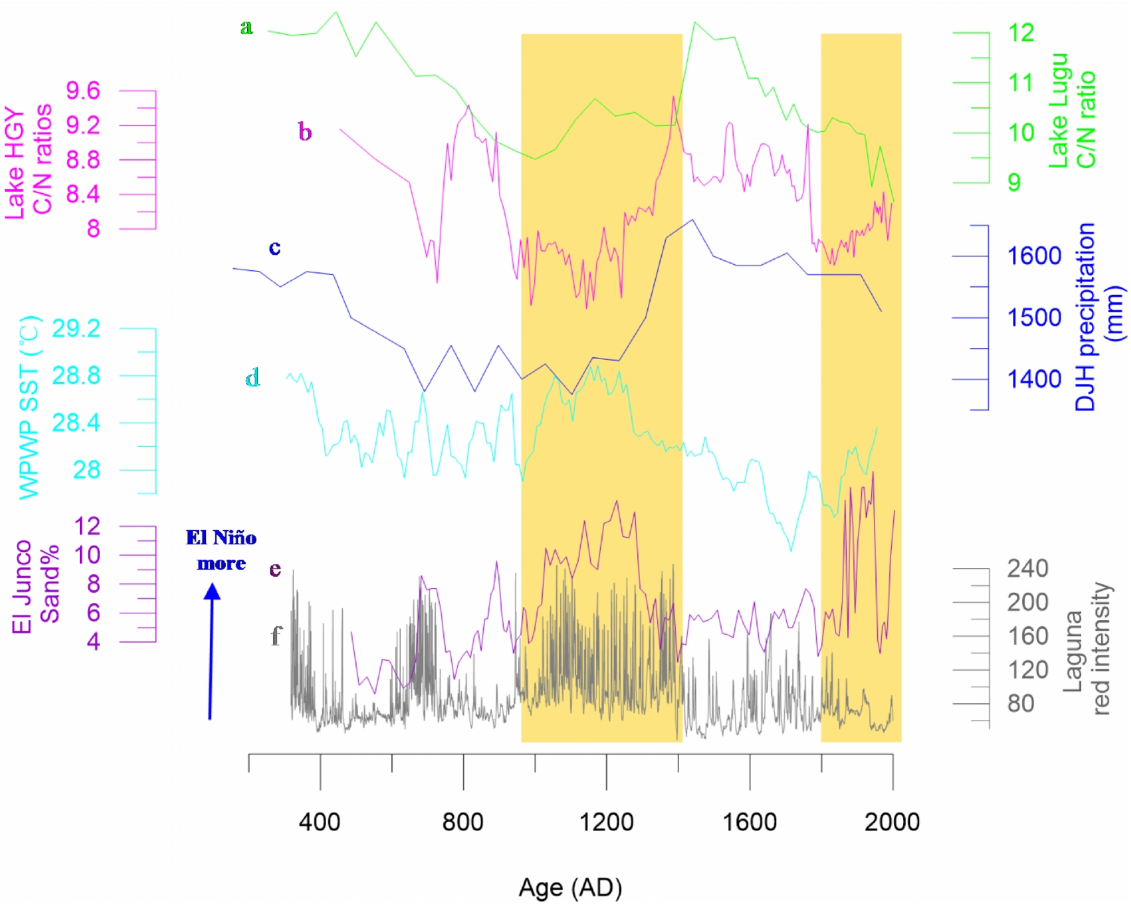

a
b
c
d
e
f
Lake HGY C/N ratios
9.6
9.2
8.8
8.4
8
WPWP SST (℃)
29.2
28.8
28.4
28
El Junco Sand%
12
10
8
6
4
El Niño more
12
11
10
9
Lake Lugu C/N ratio
1600
1500
1400
DJH precipitation (mm)
240
200
160
120
80
Laguna red intensity
400
800
1200
1600
2000
Age (AD)

Figure S6.

Table S.1. ^{14}C ages and OSL ages for samples at Lake Qinghai

^{14}C dating	Lab. No.	Sample No.	Depth(cm)	^{14}C age	Error (yr)	Median prob. (2σ) BP *	Corrected. Ages BP **	Corrected Age (AD)
Betta	\	QH10-29	29	1650	30	1551	503	1447
IEECAS	XA9302	QH10-45	45	2667	30	2773	1725	225
IEECAS	XA7775	QH10-60	60	2525	24	2615	1567	383
OSL dating	Lab. No.	Sample No.	Depth (cm)	OSL dates (AD)	Error (yr)			
IEECAS	XH140716-1	QH10-4-1	15	1880, §	30			
IEECAS	XH140716-2	QH10-4-2	45	1200	110			
IEECAS	XH140716-3	QH10-4-3	75	1430	50			

* ^{14}C ages were calibrated by Calib 6.01 [38].
** An old carbon effect of ~1048 years [39] was applied to correct the ^{14}C ages (see *refs. 1* for details).
§ OSL dating was carried out by single-aliquot regenerative-dose protocol with additional annealing step [3].

Table S.2. ^{14}C ages of the snail remains in the paleao-shorelines/profiles, and those of the living snails and modern lake waters at Lake Chenghai

Lab. No.	Sample Code	Dating Materials	pMC	error	^{14}C age	error	Cal. Age (median prob., AD) **	Elevation (m)
XA12895	CHP3-1-3	small snails	88.37	0.29	993	26	1030	1507
XA12668	CHP3-1-4	small snails	88.42	0.26	988	24	1034	1506.5
XA12807	CHP5-2	small snails	89.93	0.44	853	80	1186	1512
XA12808	CHP5-3-1	small snails	89.37	0.33	903	60	1119	1521
XA12809	CHP5-3-4	small snails	88.45	0.32	986	58	1043	1520.5
XA12819	CHP6-2-1	small snails	91.2	0.47	740	82	1266	1520
XA12820	CHP6-2-2	small snails	90.64	0.31	789	54	1242	1519.5
XA15128	CHP5-6	small snails	94.23	0.27	477	23	1432	1535.2
XA11149	CH11-1	living small snail	104.62	0.36	\	\	\	\
XA11150	CH11-2	living small snail	104.28	0.33	\	\	\	\
XA12625	CH15-1-0	* MLW: surface	102.88	0.35	\	\	\	\
XA12631	CH15-1-8	MLW: 8 m	102.49	0.32	\	\	\	\
XA12633	CH15-1-16	MLW: 16 m	102.27	0.32	\	\	\	\
XA12629	CH15-1-24	MLW: 24 m	102.94	0.30	\	\	\	\
XA12630	CH15-1-B	MLW: bottom	102.55	0.32	\	\	\	\

* MLW: modern lake water
** The ^{14}C ages were calibrated by Calib 6.01 [38.]

Table S.3. Sites mentioned in this study: the ISM region (1-20), and EASM region (21-38).

	Site No.	Site Name	Archives	Proxy indices	Climatic signal	Dating	References
ISM areas	1	Lake Turkana	Lake sediments	Carbonate content	Reconstructed lake levels	AMS^{14}C dating on the carbonate fractions	*refs.* 40,41
	2	Lake Victoria (core P2K-1)	Lake sediments	Diatom taxa (%)	Relative paleolake levels	AMS^{14}C dating on the bulk organic sediment	*ref.* 42
	3	Lake Edwards	Lake sediments	Mg% in Calcite and biogenic silica contents	drought events	AMS^{14}C dating on the terrestrial plant fragments and charcoal	*ref.* 41
	4	Lake Nyamogusingiri	Lake sediments	Diatom record	Relative lake levels	AMS^{14}C dating on the terrestrial macrofossils or charcoal	*ref.* 43
	5	Lake Kyasanduka	Lake sediments	Diatom record	Relative lake levels	AMS^{14}C dating on the terrestrial macrofossils or charcoal	*ref.* 43
	6	Lake Naivasha	Lake sediments	Sedimentology-inferred water depth; diatom taxa (%)	Lake levels; salinity	AMS^{14}C dating on the wood or macrofossils or charcoal	*ref.* 44
	7	Kilimanjaro glacier	Ice core	Dust contents	Precipitation	The 1952 time horizon, and a steady-state glacier age model	*ref.* 45
	8	Southern Oman, Qunf cave	Stalagmite, Q5	$\delta^{18}O$	Precipitation	U/Th dating	*ref.* 46
	9	Oman Gulf	Ocean sediment core	Fossil pollen and dinocyst records	vegetation types	^{210}Pb; ^{14}C dating on gastropod shells	*ref.* 47
	10	Central India, Lake Lonar	Lake sediments	multi-proxy indices	Drought/wetness; vegetation types	^{14}C dating on terrestrial wood samples	*ref.* 48
	11	Southern India, Lake Thimmannanayakanakere	Lake sediments	multi-proxy indices	lake level; paleo-rainfall	^{14}C dating on bulk organic matter	*ref.* 49
	12	Lake Lugu, S-ETP	Lake sediments	Grain size, C/N	Precipitation	^{137}Cs chronology and AMS^{14}C dating on the plant material	*ref.* 13
	13	Lake Erhai, S-ETP	Lake sediments	Pollen (%)	Precipitation	^{137}Cs and ^{210}Pb chronology, and AMS^{14}C dating on the snail shells	*ref.* 12
	14	Lake Chenghai, S-ETP	Lake shorelines/beaches	Ages of high lake levels	Lake levels	AMS^{14}C dating on the snail shells	This study
	15	Indo-Pacific warm pool	Marine sediment	$\delta^{18}O$	salinity	AMS^{14}C dating on the mixed samples of Globigerinoides sacculifer and Globigerinoides rubber, and the tephra	*ref.* 50

	16	Multi- cores from the Makassar Strait	Marine sediment	$\delta^{18}O$	salinity	^{210}Pb chronology, radiocarbon dating, and a correlation to the AD 1815 Mount Tambora ash	*ref.* 37
	17	Cattle Pond	Lake sediments	Grain size	Precipitation	^{210}Pb chronology and AMS^{14}C dating on the terrestrial organic matter and TOC	*ref.* 51
	18	Lake Huguangyan	Lake sediments	TOC, C/N, BSi	Precipitation	^{137}Cs chronology and AMS^{14}C dating on the bulk organic matter and terrestrial leaves	*ref.* 35
	19	Longgan Lake	Lake sediments	Pollen records	Precipitation	^{14}C dating on the bulk organic matter	*ref.* 52
	20	Dajiuhu	Peat sediments	MS, pollen (%)	Precipitation	^{14}C dating on peat	*ref.* 36
EASM areas	21	Qilian Mt., N-ETP	Tree rings	width	Precipitation	Tree-ring chronologies	*ref.* 14
	22	Delingha, N-ETP	Tree rings	width	Precipitation	Tree-ring chronologies	*ref.* 6
	23	Dulan, N-ETP	Tree rings	width	Precipitation	Tree-ring chronologies	*ref.* 5
	24	Lake Qinghai, N-ETP	Lake sediments	Grain size, TOC	precipitation	^{137}Cs and ^{210}Pb chronology, and OSL dating	*ref.* 1; this study
	25	Longxi Area	Historical literatures	Drought/Flood index	Precipitation	Historical literatures	*ref.* 7
	26	Wanxiang Cave	Stalagmite	$\delta^{18}O$	Precipitation	U/Th dating	*ref.* 53
	27	Huangye Cave	Stalagmite	$\delta^{18}O$	Precipitation	U/Th dating	*ref.* 8
	28	Maowusu sandlands	Dune sands	Sand-paleosol stratigraphy	Precipitation	Optically stimulated luminescence dating	*ref.* 54
	29	Lake Gonghai	Lake sediments	Magnetic parameters	Precipitation	AMS^{14}C dating on the terrestrial plant material	*ref.* 55
	30	Shihua cave	Stalagmite	Lamina thickness	precipitation	Lamina count	*ref.* 56
	31	Otindag sandlands	Dune sands	Stratigraphy, MS, grain size	Precipitation	Optically stimulated luminescence dating	*refs.* 54, 57
	32	Maili Bog	Peat sediments	Pollen (%)	Precipitation	^{14}C dating on peat	*ref.* 58

	33	Songnen sandlands	Dune sands	Sand-paleosol stratigraphy	Precipitation	Optically stimulated luminescence dating	*ref.* 54
	34	Keerqin sandlands	Dune sands	Sand-paleosol stratigraphy	Precipitation	Optically stimulated luminescence dating	*ref.* 54
	35	Korea, Seoul	Historical Literature	Drought index	Precipitation	Historical literatures	*ref.* 59
	36	Lake Nakatsuna	Lake sediments	TOC, C/N, sand (%)	Precipitation	AMS^{14}C dating on the plant material and organic matter	*ref.* 60
	37	Lake Ni-no-Megata	Lake sediments	Geochemical data	Precipitation	AMS^{14}C dating on the plant material and charcoal	*ref.* 61
	38	Lake San-no-Megata	Lake sediments	Geochemical data	Precipitation	AMS^{14}C dating on the plant material	*ref.* 61
N-India stalagmite records	39	Sahiya Cave	stalagmite	$\delta^{18}O$	Precipitation	U/Th dating	*ref.* 62
	40	Dharamjali Cave	stalagmite	$\delta^{18}O$	Precipitation	U/Th dating	*ref.* 63
	41	Wah Shikar Cave	stalagmite	$\delta^{18}O$	Precipitation	U/Th dating	*ref.* 64
	42	Jhumar Cave	stalagmite	$\delta^{18}O$	Precipitation	U/Th dating	*ref.* 64
	43	Dandak Cave	stalagmite	$\delta^{18}O$	Precipitation	U/Th dating	*ref.* 65
